# Thermodynamic control of −1 programmed ribosomal frameshifting

**DOI:** 10.1038/s41467-019-12648-x

**Published:** 2019-10-10

**Authors:** Lars V. Bock, Neva Caliskan, Natalia Korniy, Frank Peske, Marina V. Rodnina, Helmut Grubmüller

**Affiliations:** 1 Theoretical and Computational Biophysics, Max Planck Institute for Biophysical Chemistry, Am Fassberg 11, 37077 Göttingen, Germany; 2 Helmholtz Institute for RNA-based Infection Research (HIRI), Helmholtz Centre for Infection Research (HZI), Josef-Schneider-Straße 2, 97080 Würzburg, Germany; 3 Medical Faculty, University of Würzburg, Josef-Schneider-Straße 2, 97080 Würzburg, Germany; 4 Department of Physical Biochemistry, Max Planck Institute for Biophysical Chemistry, Am Fassberg 11, 37077 Göttingen, Germany

**Keywords:** Thermodynamics, Statistical methods, Translation

## Abstract

mRNA contexts containing a ‘slippery’ sequence and a downstream secondary structure element stall the progression of the ribosome along the mRNA and induce its movement into the −1 reading frame. In this study we build a thermodynamic model based on Bayesian statistics to explain how −1 programmed ribosome frameshifting can work. As training sets for the model, we measured frameshifting efficiencies on 64 *dnaX* mRNA sequence variants in vitro and also used 21 published in vivo efficiencies. With the obtained free-energy difference between mRNA-tRNA base pairs in the 0 and −1 frames, the frameshifting efficiency of a given sequence can be reproduced and predicted from the tRNA−mRNA base pairing in the two frames. Our results further explain how modifications in the tRNA anticodon modulate frameshifting and show how the ribosome tunes the strength of the base-pair interactions.

## Abstract (web summary)

Programmed ribosomal frameshifting (PRF) is an alternative translation strategy that causes controlled slippage of the ribosome along the mRNA, changing the sequence of the synthesized protein. Here the authors provide a thermodynamic framework that explains how mRNA sequence determines the efficiency of frameshifting.

## Introduction

Ribosome frameshifting is a recoding event that causes ribosome slippage along the mRNA and changes the sequence of the synthesized protein. Frameshifting can direct the ribosome into −1, +1, −2, or even +4 frame, but the most common type is −1 frameshifting^1–4^. Spontaneous ribosome slippage is a rare event that occurs, on average, once in 10^4^–10^**5**^ codons^5,6^. This low spontaneous frameshifting increases dramatically on particular mRNAs that contain sequences for programmed ribosomal frameshifting (PRF). PRF requires a slippery sequence, which usually comprises a X XXY YYZ heptamer, where XXX and YYY are triplets of identical bases and Z is any nucleotide, which allows for cognate pairing of the P-site and A-site tRNAs in the 0-frame and −1-frame^7–9^. The nature of the tRNAs bound to the slippery site codons is critical, including the modifications of nucleotides in the anticodon loop (i.e., at positions 34 and 37 of the tRNA)^8,10,11^. Frameshifting is facilitated by mRNA elements that slow down the ribosome progression along the mRNA, such as secondary structures (stem loops or pseudoknots) downstream of the slippery site. −1PRF is common in viruses and is found also in bacteria and eukaryotes^12–14^, where it is employed to increase the coding capacity of the genome, define stoichiometry of translation products or to regulate the lifetime of the mRNA.

There are several possible pathways that can lead to −1PRF^15^. Ensemble kinetics and smFRET studies suggested that −1PRF occurs predominantly during the translocation step when the two tRNAs in the A and P sites move to the P and E sites, respectively^16–21^. Displacement of the tRNAs weakens the interactions with the mRNA and the ribosome^22^. The mRNA secondary structure element downstream of the slippery site impedes the reverse movement of the 30S ribosomal subunit head domain, which resets the ribosome interactions with the mRNA–tRNA complex in the P site^19^. While the ribosome makes multiple attempts to complete translocation, tRNAs can realign into the −1-frame^17,18^. Alternatively, frameshifting can occur when the delivery of aminoacyl-tRNA to the A site is delayed, e.g., due to the lack of a particular amino acid^20,21,23,24^. In this case, a tRNA bound to the P site can slip from the 0-frame to the −1-frame independently of the mRNA secondary structure elements^20,21^.

The best characterized example of −1PRF is *dnaX* from *Escherichia coli* containing a slippery sequence A_1_ AAA_4_ AAG_7_ (numbers denote the nucleotides within the slippery sequence), a downstream stem-loop element, and an additional stimulatory Shine–Dalgarno (SD)-like sequence upstream of the slippery sequence. The frameshifting efficiency (FS) on *dnaX* is 70–80% in vitro and in vivo^16,20,25,26^. Both codons of the slippery sequence are read by tRNA^Lys^. *E. coli* has a single tRNA^Lys^ isoacceptor (anticodon ^3′^UUS^5′^), for decoding the two Lys codons, AAG and AAA. The modified nucleotide mnm^5^s^2^U (S) at the first anticodon position (U34 in the tRNA sequence) is important for the wobble base pairing at the third codon position^27^. The modified nucleotide base pairs more stably with A than with G (ref. ^28^), which may favor tRNA^Lys^ slippage on A_4_ AAG_7_ codons^8^. In *E. coli*, also nucleotide A37 adjacent to the anticodon of tRNA^Lys^ is modified. The modified nucleotide, t6A, forms cross-strand stacking interactions with the A in the first codon position and thereby stabilizes weak A–U base-pair interactions in the first codon position^27,29^.

Structural and mutational studies suggest that tRNA modifications and the extended wobble propensity of the tRNA affect −1PRF^8,30^, yet the mechanism behind this effect is not well understood. Mutations in the A_1_ AAA_4_ AAG_7_ slippery sequence reduce frameshifting, e.g. replacing the A at position 3 or 6 (A_1_ AGA_4_ AAG_7_ and A_1_ AAA_4_ AGG_7_) diminish −1PRF to background levels^20,26^. However, in some cases the results of mutational studies are difficult to rationalize. For example, mutations of the first slippery codon A_1_ or A_4_ to G or U, which also disrupt the slippery sequence and should disfavor the codon–anticodon interaction in the −1-frame, reduce FS from 80% to 30–40%, but do not abolish frameshifting^26^. The interpretation of mutational data and understanding the frameshifting efficiencies at other slippery sequences is hampered by the lack of a conceptual model for −1PRF that could quantitatively predict frameshifting efficiencies for various tRNAs and slippery sequences.

In this work, we show that the FS can be quantitatively explained by a simple thermodynamic model based on the free-energy difference of the mRNA–tRNA base pairing between the 0-frame and the −1-frame. We used Bayesian statistics to construct and test such a model using measured FSs for 64 variants of slippery sequences containing Lys (AAA/G) and Phe (UUU/C) codons. The free-energy differences of tRNA base pairing in the 0-frame and −1-frame enabled us to reproduce FS for mRNA sequences used in the model and to predict efficiencies for sequences that were not used in the model. The model explains why A_1_G and A_4_G mutations of the A_1_ AAA_4_ AAG_7_ motif support surprisingly efficient frameshifting, shows how synonymous codons affect FS, and suggests how tRNA modifications contribute to frameshifting. Furthermore, comparison of the base-pair free-energy differences on the ribosome and in solution allows us to quantify the effect of the ribosome environment on the stability of the codon–anticodon complexes. These results explain the results of previous mutational and biochemical studies^16,18,20,26,31^ and suggest that the frameshifting efficiency is controlled mainly by the relative thermodynamic stability of the codon–anticodon interactions in the 0-frame and −1-frame.

## Results

### Experimental estimation of FS

**Fig. 1:**
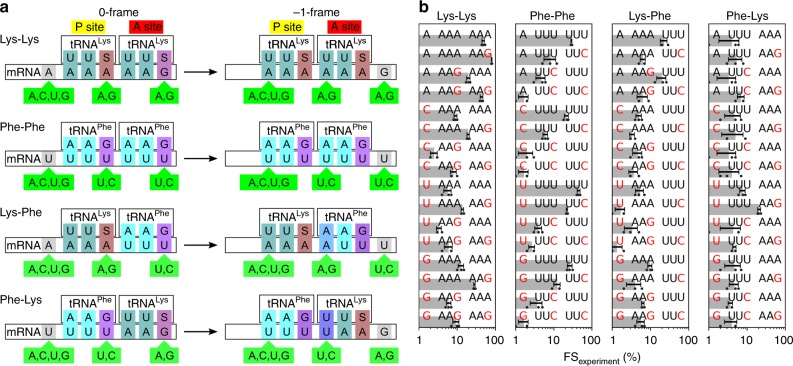
Variants of the *dnaX* slippery sequence. **a** Mutations of the slippery sequence coding for Lys–Lys, Phe–Phe, Lys–Phe, and Phe–Lys. For each tRNA pair (upper row), the mRNA sequence (lower row) is shown for the 0-frame (left) and the −1-frame (right) together with mutations (light green) that do not change the codon identity in the 0-frame, but affect frameshifting. The resulting codon–anticodon interactions at the slippery site codons are highlighted by colors, with Watson–Crick interactions highlighted in light and dark cyan; G·S and A·S pairs, where S denotes the modified nucleotide mnm^5^s^2^U (Supplementary Fig. 4), in dark purple and brown, respectively; the U·G wobble pair in purple; and A·A and U·U mismatches in different shades of blue. **b** FS (gray bars) for the indicated slippery sequence variants. Error bars denote the standard deviation from at least three independent experiments (dots, *N* **≥** 3, Supplementary Table 1)

We first compiled an experimental dataset of FS values using the established in vitro approaches^19,20^ (see the “Methods” section). We used the *dnaX* mRNA fragment encoding the slippery sequence Lys_1_Lys_2_ flanked by an upstream SD-like sequence and a downstream stem-loop structure. We generated all possible mutant variants that encoded for combinations of either Lys or Phe codons in the 0-frame (Fig. 1, Supplementary Fig. 1a, and Supplementary Table 1). 70S ribosomes programmed with *dnaX* mRNA and carrying the initiator f[^3^H]Met-tRNA^fMet^ in the P site were mixed with aminoacyl-tRNAs that are required to translate the mRNA up to the frameshifting site (one of which is [^14^C]-labeled) and elongation factors EF-Tu and EF-G. Translation products of the 0-frame and −1-frame were separated by reversed-phase high-performance liquid chromatography (RP-HPLC) and quantified by scintillation counting (Supplementary Fig. 1b). FS was calculated as a ratio of the −1-frame product to the sum of 0-frame and −1-frame peptides (Fig. 1b, Supplementary Figs. 2b, c,  3b, c, Supplementary Table 1).

We first validated that mutations in the slippery sequence do not alter the mechanism of frameshifting using the codon-walk approach^20^. Previous results indicated that the rate of Lys_1_ and Lys_2_ incorporation on slippery codons AAA_4_ and AAG_7_ is independent of the slippage-inducing mRNA stem-loop, whereas incorporation of the subsequent Phe (F) is delayed 20-fold by the stem-loop^20^. This indicated that the ribosome is stalled after the incorporation of Lys_2_ on the AAG_7_ codon and that frameshifting takes place during the delayed translocation. The incorporation of Val in the −1 frame is very efficient compared to the 0-frame Phe and the FS calculated from the ratio of the rate constants of −1 frame to 0-frame Val and Phe incorporation, respectively, is consistent with the end-point measurements (Supplementary Fig. 1b).

To test whether the branch point of frameshifting is the same on other slippery site variants, in particular those where frameshifting involves non-Watson–Crick codon–anticodon interactions in the −1-frame, we determined the rates of frameshifting for three slippery sequence variants, A_1_G, A_1_U, and A_4_G. With these mRNAs, the presence of the downstream stem-loop did not affect the incorporation rate for Lys_2_, but reduced the rate of the 0-frame Phe incorporation. This indicated that the point of slippage remains unchanged with the mRNA variants (Supplementary Figs. 2d, e,  3d, Supplementary Table 2). Analogous to the A_1_ AAA_4_ AAG_7_ slippery sequence, A_1_G, A_1_U, and A_4_G mRNA variants maintained very efficient frameshifting as shown by the high rate of Val incorporation (Supplementary Figs. 2b, c,  3b).

Another observation suggesting that frameshifting takes place at the translocation step is that with the wild-type *dnaX* sequence, the presence or absence of tRNAs decoding the Phe (0-frame) or Val (−1-frame) codons downstream the slippery sequence does not change the FS. This indicates that the commitment to the new reading frame occurs before the Val or Phe codon is available for decoding in the A site, that is at the preceding translocation step^20^. Similarly, for the A_1_G, A_1_U and A_4_G variants the incorporation efficiency is not affected by the presence of Val-tRNA and Phe-tRNA encoding the overlapping (G UUC) codons (Supplementary Figs. 2b, c and 3b). For the A_4_G construct, if tRNA slippage occurred on the first codon (AAG_4_) while the second codon was free, the second codon would be decoded in the −1-frame (G_4_AA) by Glu-tRNA^Glu^, resulting in a MAKE peptide. However, in the presence of Glu-tRNA^Glu^, the MAKK peptide is still predominant (Supplementary Fig. 3b), indicating that slippage takes place after the decoding of the second codon by tRNA^Lys^. In the presence of all aa-tRNAs, we observe only about 5% of the frameshifting peptide containing Glu (MAKEV) (Supplementary Fig. 3d), rendering an alternative frameshift pathway, e.g., stimulated by aa-tRNA depletion, unlikely under given conditions.

We then replaced the wild-type *dnaX* slippery Lys_1_Lys_2_ codons with all possible sequences that encode synonymous Lys (AAA/G) or Phe (UUU/C) codons: tRNA^Lys^ and tRNA^Lys^, tRNA^Phe^ and tRNA^Phe^, tRNA^Lys^ and tRNA^Phe^, or tRNA^Phe^ and tRNA^Lys^ (Fig. 1a, Supplementary Table 1). For all codons in the 0-frame of these sequences, base pairs in the first and second positions of the codon–anticodon complex are only Watson–Crick base pairs, whereas in the third position Watson–Crick and wobble base-pairs are tolerated^27,32^. The FS of the wild-type slippery sequence is about 80%, consistent with the previous reports in vivo and in vitro^16,20,25,26^. Several slippery-sequence variants also support efficient frameshifting, whereas others diminish frameshifting considerably. Notably, for sequences that have exactly the same codons in the 0-frame and −1-frame (A_1_ AAA_4_ AAA_7_ and U_1_ UUU_4_ UUU_7_) the FS is very close to 50% (Fig. 1b), suggesting that the 0-frame and −1-frames are equally possible at these conditions, i.e., the ribosome is not inherently committed to maintaining the 0-frame. The FS was high not only with the sequences where −1PRF resulted in canonical Watson–Crick base pairs in the −1 reading frame, but also in several cases with the expected first position mismatch on one of the slippery codons, such as for C/U/G_1_ AAA_4_AAA_7_, A_1_AAG_4_AAA/G_7_, A/C/G_1_ UUU_4_ UUU_7_, A_1_ AAG_4_ UUU_7_ or U_1_ UUU_4_ AAG_7_ (the −1-frame codon is underlined). Even with two first position mismatches, such as with G/C_1_ AAG_4_ AAG_7_ the FS is 8–9%, well over the minimum FS value of about 2% found for this tRNA pair on the C_1_ AAG_4_ AAA_7_ sequence.

### Free-energy model of −1PRF

Upon frameshifting, the mRNA–tRNA base pairs change depending on the slippery sequence (Fig. 1a). We asked whether the FSs can be explained by differences in the interaction free-energy between the base pairs involved in the 0-frame and −1-frame duplex. If all individual base-pair free-energy differences Δ*G*_bp_ were known, and assuming thermodynamic equilibration during the frameshifting, the FS of a given mRNA sequence could be calculated from the total free-energy difference Δ*G* for all anticodon–codon positions in the 0-frame and −1-frame, FS = exp(−Δ*G*/(*k*_B_*T*))/[1 + exp(−Δ*G*/(*k*_B_*T*))] with Boltzmann factor *k*_B_ and temperature *T* = 310 K. However, initially we face the inverse problem, as the FS values were known from experiments (Fig. 1b), whereas the base-pair free-energy differences were unknown. To tackle this inverse problem, we used Bayesian statistics to obtain the individual free-energy differences that best fit all measured FSs (see the “Methods” section).

The ribosome provides structurally different environments for the mRNA–tRNA interactions at the first and the second slippery codons, which likely has an effect on the base-pair free energy^27,33–35^. To take into account this effect of the different environments on the base-pair free energies Δ*G*_bp_, we used 16 Δ*G*_bp_ variables for all base-pair changes at all position of each codon, of which the 14 Δ*G*_bp_ variables turned out to be independent. Although frameshifting occurs in the intermediate state of translocation, most likely in the ap/P–pe/E chimeric hybrid state^22^, for ease of notation we indicate the interactions for the first and second codon as P and A, respectively. The position of a base pair (X·Y) within the codon is indicated by a number (1, 2, 3) and the first base (X) denotes the codon base and the second the anticodon base (Y).

**Fig. 2:**
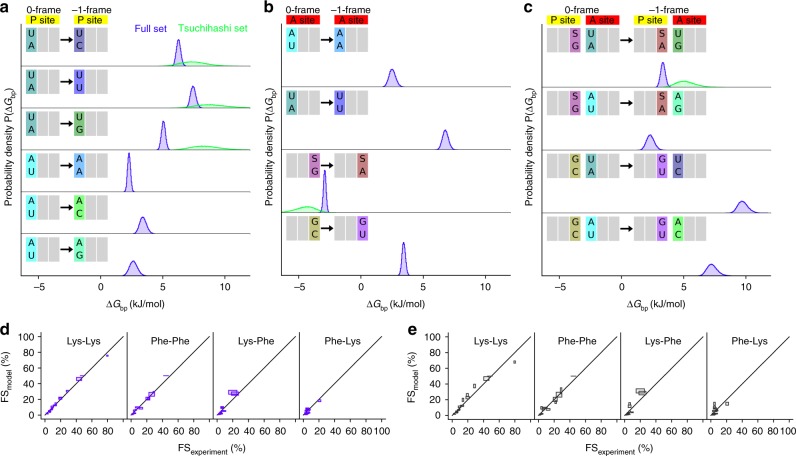
Inferred mRNA–tRNA base-pair free-energy differences on the ribosome during frameshifting. **a** Probability densities (colored histograms) of the free-energy differences Δ*G*_bp_ for the P-site base pairs obtained from the model based on the full FS data set (64 FS values, blue), and a set of the FS values measured in vivo by Tsuchihashi et al.^26^ (21 FS values, green). **b** Probability densities for changing A-site base pairs. **c** Probability densities for changing P-site and A-site base pairs simultaneously. **d** FS from experiment compared to that calculated from the free-energy model. Each panel shows FS values for a tRNA pair (rmsd 2.5%). **e** For cross-validation of the model, iteratively, each FS value was predicted using free-energy differences obtained from all other FS values (rmsd 4.1%). For each mRNA sequence, one square is centered at the mean value and the width and height correspond to two times the standard deviation

First, we tested whether the free-energy model with the underlying assumptions and the set of parameters is able to reproduce the measured FS values. To that end, we used all 64 measured FS values (Fig. 1b) to obtain base-pair free-energy differences for 10 single base-pair changes in the P site (Fig. 2a) and in the A site (Fig. 2b). The remaining six base-pair changes only occur in pairs in the sequences used here (Fig. 2c). For these changes, we obtained the free-energy differences of changing two base pairs at the same time. Positive free-energy differences indicate that the free energy of base pairing in the −1-frame is larger than in the 0-frame, i.e., that base pairing in the −1-frame is less favorable than in the 0-frame. Accordingly, the larger free energy of the −1 frame renders frameshifting less likely, and thus FS < 50% is expected. Next, we calculated distributions of FS values (FS_model_) from the obtained distributions of the free-energy differences using the ratio of Boltzmann distributions shown above (see the “Methods” section). Despite the fact that our model only contains 14 free parameters, the calculated efficiencies agree well with the measured efficiencies with a root mean square deviation (rmsd) of 2.5% between the mean FS_model_ and the FS_experiment_ (Fig. 2d).

Second, we tested if the free-energy model is also able to predict FS values for mRNA variants that were not used to obtain the free-energy differences. To this aim, we omitted one mRNA variant from the dataset and derived a new free-energy model in terms of a complete set of free-energy differences. This set was subsequently used to predict the efficiency of the omitted variant. We repeated this cross-validation procedure for each mRNA variant and good agreement with the measured efficiences was seen (Fig. 2e, rmsd 4.1%), underscoring that the model is indeed predictive.

**Fig. 3:**
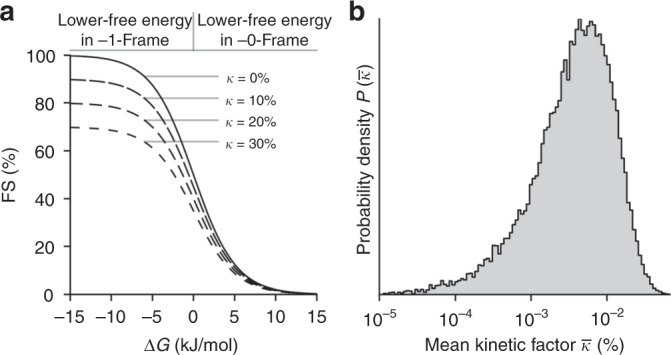
Kinetic contribution to the frameshifting efficiency. **a** FS as a function of the free-energy difference Δ*G* between 0-frame and −1-frame for different kinetic factors *κ*, where *κ* = 0% corresponds to frameshifting in equilibrium. **b** Probability density for the mean kinetic factor, obtained from a modified free-energy model that takes into account kinetic barrier crossing between 0-frame and −1-frame

One critical assumption underlying our thermodynamic equilibrium model is that the FS is determined only by the free-energy difference of tRNA binding in −1-frame vs. 0-frame. This is only possible if frameshifting is significantly faster than the completion of translocation. In such a case, the kinetic partitioning between frameshifting and translocation would be negligible and the probability of the ribosome to switch into −1-frame would be predominantly thermodynamically controlled. This assumption is plausible, because translocation is slowed down considerably by the mRNA stem-loop, but as the elemental rate of tRNA slippage is not known, the extent to which frameshifting is affected by kinetics is unclear. To challenge this assumption, we included a kinetic factor *κ* into our model such that FS_kinetic_ = FS · (1−*κ*), where FS is obtained from the ratio of Boltzmann distributions (see the “Methods” section; Fig. 3a). For the kinetic factor, we assumed that a free-energy barrier limits the rate of tRNA slippage from the 0-frame to the −1-frame and back, i.e. the slippage is slower than translocation. The mean kinetic factor for all mRNA variants was included within our Bayesian approach as a nuisance parameter. Similar as above, the probability of base-pair free-energy differences was obtained for all 64 sequences, now additionally including *κ*. The resulting rmsd value (2.5%) is similar to that from the model without the kinetic contribution. Further, the most probable mean kinetic factor *κ* was found to be below 0.1% (Fig. 3b), which indicates that the kinetics of frameshifting does not markedly affect the FS in this experimental system. Independent estimations of frameshiting kinetics suggest that the rate of slippage into −1-frame is 10 s^−1^ on the original *dnaX* slippery sequence and 3 s^−1^ on the A_4_G sequence (B.-Z. Peng, L. V. Bock, R. Bellardinelli, F. Peske, H. Grubmüller, and M. V. Rodnina, unpublished data). In comparison, the step that limits the completion of translocation was estimated to 0.1–0.5 s^−1^ (refs. ^19,20^). This supports the notion that, with the frameshifting secondary structure element on the mRNA, completion of translation is sufficiently slow to allow the tRNAs to re-pair with their thermodynamically favored codons.

In summary, these results show that the FS values are consistent with—and can even be predicted by—a thermodynamic model that is based on only two assumptions, (1) that all FS values are determined solely by the free-energy differences between the 0-frame and −1-frame and (2) that the total free-energy difference is the sum of the individual free-energy differences for each mRNA–tRNA base-pair change upon frameshifting, i.e., that the coupling of free-energy changes of base pairs is small.

### Free-energy model applied to an independent dataset

**Fig. 4:**
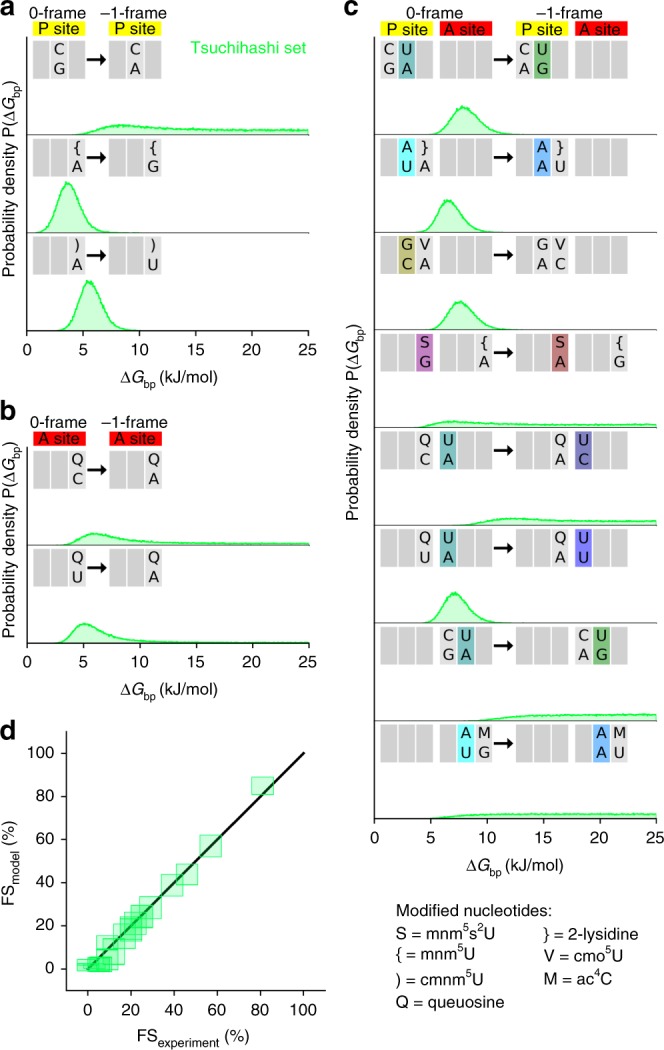
mRNA–tRNA base-pair free-energy differences obtained from published FS values measured in vivo^26^. **a**–**c** Probability densities of the free-energy differences Δ*G*_bp_ upon changing base pairs in the P site, the A site, as well as in the P and A sites simultaneously, obtained from the free-energy model applied to the Tsuchihashi FS set. **d** FS values from the Tsuchihashi set compared to those calculated from the model (rmsd 2.6%). For each mRNA sequence, one square is centered at the mean value and the width and height correspond to two times the standard deviation

Tsuchihashi et al. reported a large set of FS values for 23 variants of the *dnaX* mRNA in vivo^26^, which enabled us to test the free-energy model of frameshifting against an independent dataset. The alternative dataset by Sharma et al.^36^ could not be used, as the values for FS on the native *dnaX* slippery sequence are inconsistent with those of refs. ^20,26^, thereby precluding meaningful comparisons. We used 21 FS values from the Tsuchihashi dataset (Supplementary Table 3) to obtain probabilities for free-energy differences, excluding the G_**7**_A variant, which was considered unreliable by the authors due to the possibility of transcriptional slippage^26^, and the 123C variant which encodes a proline and may reflect proline-specific stalling effects^37^. The 21 mRNA variants not only include seven variants that have been tested in this work, but also sequences that do not preserve the Lys or Phe codon identity, because their P-site and A-site codons encode different amino acids in the 0-frame. As a consequence, a larger set of 26 individual base-pair changes upon −1-frameshifting can be considered in the model. Notably, we did not include any information about the experimental FS values obtained in this work (Fig. 1b), so using the Tsuchihashi dataset provides an independent test for the model in vivo. The green histograms in Fig. 2a–c show the probability distributions for the free-energy differences obtained from both datasets, and Fig. 4a–c show the distributions for the remaining base-pair changes. We obtained free-energy differences for six base-pairs in the P site (Figs. 2a and 4a) and for three in the A site (Figs. 2b and 4b), as well as for nine combinations of P-site and A-site base pairs (Figs. 2c and 4c).

The FS values calculated from the free-energy differences are in excellent agreement with the measured FS values, with an rmsd of 2.6% (Fig. 4d). Notably, the probability distributions obtained using the Tsuchihashi et al. dataset are much broader than those obtained from our data set (Fig. 2a–c). For some base-pair changes, the probability density extends towards large free-energy differences, indicating that FS values only provide a lower boundary, e.g., for P2 G·C → A·C (Fig. 4a, top panel). A lower boundary arises from the close to 0% FS values, for which the corresponding ΔG values increase without limit, such that no upper boundary is obtained. Overall, the broader distributions reflect a higher uncertainty of the free-energy differences obtained from the Tsuchihashi et al. data set than to those derived from our dataset—mostly due to the smaller number and larger experimental uncertainties of the FS values used to obtain free-energy differences for a larger number of base-pair changes.

### Free-energy differences of base-pair changes

As expected, all free-energy differences Δ*G*_bp_ for changing a Watson–Crick base pair (A·U or U·A) into a mismatched base pair (C·U, U·U, G·U, A·A, C·A, G·A) are positive (Fig. 2a, b, Supplementary Table 4). Following this notion, more mismatches introduced in the −1 frame imply lower FS. Interestingly, changing the Watson–Crick A·U base pair to pyrimidine·pyrimidine (C·U or U·U) base pairs comes with the highest energetic penalty, whereas changing the Watson–Crick base pair to a mismatched purine·pyrimidine base pair (A·U → G·U) or to a mismatched pyrimidine·purine (U·A → C·A) has a lower penalty. The lowest penalty comes with the change to purine·purine base pairs (A·A or G·A). It is possible that in the codon–anticodon helix two pyrimidines are too far apart, while a pair of larger bases can form contacts, albeit not as well as the Watson–Crick base pairs. For the sequences investigated here, the only base-pair change with a negative Δ*G*_bp_ is G·S to A·S in the wobble position of the A-site codon (A3). The nucleotide S is a modified U (mnm^5^s^2^U) which interacts more strongly with A than with G; Δ*G*_bp_ = −2.9 or −4.6 kJ/mol from our in vitro dataset and the Tsuchihashi et al. dataset, respectively (Fig. 2b, Supplementary Table 4). For the similar modified nucleotide mnm^5^U, which lacks the s^2^ modification, the interaction with A is also stronger than with G and the free-energy difference Δ*G*_bp_ is similar, −3.8 kJ/mol (Fig. 4a, Supplementary Table 4; note that the positive values shown therein are for the A → G change, whereas in Fig. 2b the change is in opposite direction, G → A). The similar free-energy differences of mnm^5^s^2^U and mnm^5^U suggest that the s^2^ modification of U does not play a large role in the base-pair free energy. This is different with yeast tRNA^Lys^, where mcm^5^-modified tRNA^Lys^ lacking the s^2^ group has a lower affinity of binding to the cognate codon AAA than the fully modified tRNA^Lys^^38^. The difference may be related to the existence of two tRNA isoacceptors in yeast dedicated to reading of AAA or AAG codon each, whereas in *E. coli* one tRNA isoacceptor reads both codons.

tRNA^Phe^ with the ^3′^AAG^5′^ anticodon is able to decode codons UUC and UUU through C·G Watson–Crick or U·G wobble base pairing at the A3 codon position. The free-energy difference between the two base pairs (A3 C·G → U·G) is 3.4 ± 0.1 kJ/mol (Fig. 2b, Supplementary Table 4) which agrees well with the values of 6.2 ± 3.0 or 1.3 ± 2.5 kJ/mol obtained from free-energy molecular-dynamics simulations with two different starting structures^35^. The nucleotide queosine (Q) is a modified G. Changing C·Q to A·Q or U·Q to A·Q has similar free-energy costs (Fig. 4b), indicating that the base-pair free energies of C·Q and U·Q are similar. Queosine is found in the ^3′^UUQ^5′^ anticodon of tRNA^Asn^ which decodes both AAC and AAU codons. In agreement with the similar free energies, FS is similar on U_1_ UUA_4_ AAC/U_7_ mRNA variants (2% and 3%)^39^. In the absence of the modification (^3′^UUG^5′^ anticodon), the FS of the AAC_7_ variant is lower (1%) than for the AAU_7_ variant (5–6%)^39^, as expected from the result that the U·G wobble base pair is weaker than the C·G base pair.

The obtained free-energy differences also explain the unexpected observation that mutations A_1_G and A_4_G retain a surprisingly high FS despite the fact that they involve a mismatch in the −1-frame. The A_1_G sequence (G_1_ AAA_4_ AAG_7_) undergoes two base-pair changes upon shifting to the −1-frame. The first change introduces an unfavorable mismatch in the first position of the first codon, P1 A·U → G·U, which comes with a free-energy penalty of 5.1 kJ/mol (Supplementary Table 4, Δ*G*_bp_ obtained from our dataset). The second base-pair change, the A3 G·S → A·S at the A-site wobble position, however, reduces the free-energy difference by −2.9 kJ/mol. Therefore, the total energetic cost Δ*G* between the frames is only 2.2 kJ/mol, which, using the ratio of Boltzmann probabilities, results in a FS of 30% that is close to the measured value of 28%. The sequence of A_4_G (A_1_ AAG_4_ AAG_7_) also introduces one G·U mismatch (A1 position) upon frameshifting, but has an even higher measured FS of 44%. Our model attributes this higher FS to a favorable G·S → A·S base-pair change in the A3 position (−2.9 kJ/mol), which almost neutralizes the unfavorable changes in P3 and A1 (G·S → A·S and A·U → G·U, respectively) of 3.3 kJ/mol to a total free energy cost Δ*G* = 0.4 kJ/mol, which results in a FS of 46% in agreement with the measured value of 44%.

**Fig. 5:**
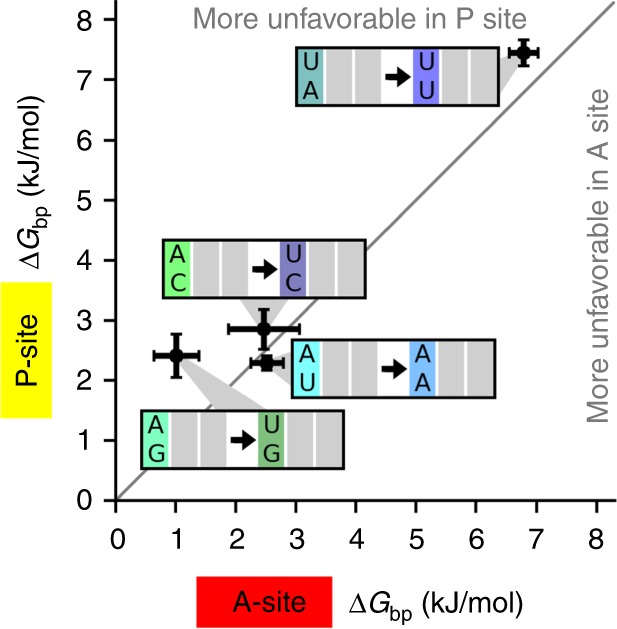
Comparison of P-site and A-site base pairs. Free-energy differences for the A and the P sites shown as the mean (points) and standard deviations (error bars)

The environment of the codon–anticodon base pairs is different in the P and A sites, which prompted us to compare the individual free-energy differences in the P and A sites, respectively. The free-energy differences for A·U → U·U and U·A → A·A, which have been obtained directly (Figs. 2a, b and 5), are similar in the P and A sites. The free-energy differences for C·A → C·U and G·A → G·U were not obtained directly, but inferred from a combination of free-energy differences under the assumption that the A·U and U·A base pairs have the same free energy (see the “Methods” section). The resulting free-energy differences for C·A → C·U are similar in the P and A sites (Fig. 5). The G·A → G·U change appears somewhat less favorable in the P site than in the A site by about 1 kJ/mol, but this difference could also arise from a non-isostericity of the A·U base pair. Overall, these results suggest that the base-pair free energies in the first position of the codon–anticodon helix are similar in the P-site and A-site environment.

### Base-pair interactions in solution and on the ribosome

**Fig. 6:**
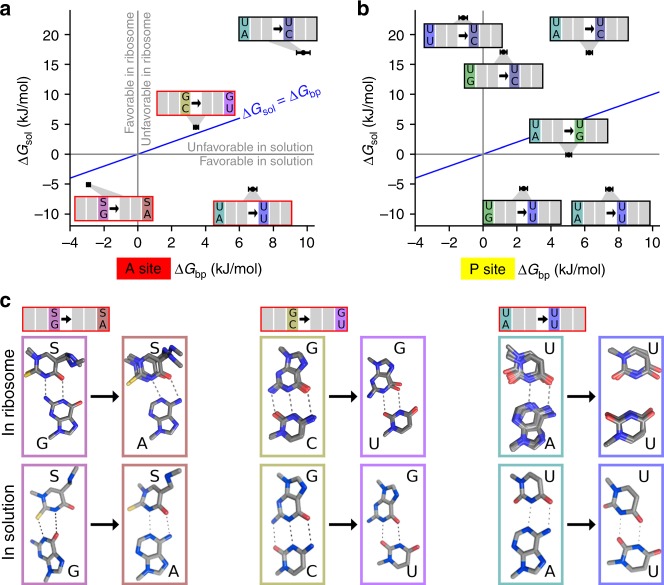
Base pairs in solution and on the ribosome. **a** In the A site. Free energy of base pairs in solution, Δ*G*_sol_, was estimated from MD simulations^40^. For Δ*G*_bp_ values, the mean (circle) and standard deviation (bar) of the probability densities is shown (from Fig. 2a). Gray lines (at Δ*G* = 0) indicate whether a given base pair change is favorable or unfavorable. Blue line separates the regions where the interaction is more favorable on the ribosome (above the line) or in solution (below). **b** In the P site. **c** Upper row, base-pair conformations in the A site from X-ray structures^27,34^. Lower row, the minimum free-energy conformation for the same base pairs in solution (adapted from Vendeix et al. ^40^)

To further investigate the effect of the ribosome on the mRNA–tRNA base pairs, we compared the free-energy differences of the base pairs obtained from our model, Δ*G*_bp_, with those calculated from free-energy molecular dynamics simulations for isolated base pairs in solution, Δ*G*_sol_^40^ (Fig. 6a, b). The conformations of several Watson–Crick and mismatched mRNA–tRNA base pairs have been obtained by X-ray crystallography^27,34^, which allows us to check whether the predicted free-energy differences are reflected in the conformational differences. The G·S → A·S change is favorable both in solution and in the A1 codon position on the ribosome, but it is slightly more favorable in solution (Fig. 6a). The G·S and A·S base pairs both have two H-bonds on the ribosome^27^ and in solution^40^, but the base-pair conformations differ (Fig. 6c, left panel), which agrees well with the different free-energy differences. Interestingly, the sulfur atom (yellow) of the modified nucleotide mnm^5^s^2^U (S) is not directly involved in the base pairing on the ribosome, which also agrees with the observation that mnm^5^U, which lacks the sulfur atom, shows similar free-energy differences as mnm^5^s^2^U (see above).

Base-pair changes introducing mismatches C·G → U·G and A·U → C·U are unfavorable both in solution and on the ribosome (Fig. 6a). For the C·G → U·G change, the A-site conformations of the base pairs^34^ are similar to those in solution (Fig. 6c, middle panel) and indicate a loss of one H-bond, which again agrees with the positive free-energy differences Δ*G*_bp_ and Δ*G*_sol_. In contrast, the A·U → U·U base-pair change is favorable in solution, but unfavorable on the ribosome (Fig. 6a). This result agrees with the structural data which shows that for the U·U base pair a Watson–Crick-like conformation is enforced in the A site, which prevents H-bond formation^27^, while in solution two H-bonds can form, stabilizing the base pair (Fig. 6c, right panel).

In the P site, base-pair changes to U·U are favorable in solution, but unfavorable on the ribosome, which suggests that also in the P site the favorable non-Watson–Crick conformation is prevented (Fig. 6). The base pairs A·U and G·U have similar free energies in solution and the G·U engages in a non-Watson–Crick conformation^40^. In the P site, in contrast, the G·U base pair is predicted to be weaker than the A·U base pair, which agrees with the observation that Watson–Crick-like conformation are also enforced in the P site^41^. Overall, these comparisons indicate that the predicted free-energy differences agree with qualitative expectations solely based on structural studies, thus providing further and independent support for our free-energy model.

## Discussion

The present work provides the thermodynamic framework for understanding PRF. We show that, when translocation is slowed down by an mRNA secondary structure element downstream of the slippery site, the propensity of a tRNA to frameshift on a given slippery codon is largely determined by the free energies of base pairing, and the FS can be confidently predicted based solely on the combinations of base pairs at a given frameshift site. For the presented system, kinetic effects do not contribute to frameshifting, indicating that the stalling time is sufficiently long to allow equilibration via frequent re-crossings of the free-energy barriers between the two reading frames. As a result, only the free-energy difference between the frames governs the FS. The long pause of the ribosome at an mRNA secondary structure element may serve to achieve sufficient equilibration and thereby contribute to enhance the FS. For shorter stalling times, the pause would become too short for the barrier to be overcome sufficiently often, and thus one would predict the FS to decrease with increasing translocation rate. In this non-equilibrium case, the free-energy model that includes the kinetic contribution should provide good estimates for the rates of recoding. It would be interesting to see whether these rates differ for different tRNA species or for modified vs. non-modified tRNAs, which would indicate that certain tRNAs in the cell are more prone to frameshifting.

Our model also shows that for the studied mRNA variants under the given conditions, the free-energy differences of the individual base-pair changes are additive and appear not to be energetically coupled. The observation that the decoding center is in the same conformation with bound cognate or near-cognate tRNAs suggests that the free-energy difference between the two cases stems from the base-pair interactions^27,34,41,42^, providing a possible reason for the absence of coupling. In all of the studied mRNA variants, the base pairs in second codon positions remain unchanged upon frameshifting (Fig. 1a). However, if base pairs at the first and second codon positions would be changed at the same time, the helix conformation might change markedly, rendering a coupling between base-pair changes more likely. This effect can be tested by applying the free-energy model to a larger set of FS values for mRNA variants including sequences that lead to position 1 and 2 mismatches. Furthermore, the Watson–Crick-like conformations of U·G mismatches^34,41,42^ imply that the free-energy difference of changing a C·G to U·G in the first two codon positions is different from that in the third positions, where wobble base pairs are tolerated^34^. It was suggested that either tautomerization or ionization of the bases allow the U·G base pairs to adopt the Watson–Crick-like conformation^41^. Our free-energy model applied to frameshifting efficiencies of mRNA variants which entail the C·G to U·G base-pair changes in different codon positions would provide access to the corresponding free-energy differences.

In summary, the thermodynamic model of frameshifting can be applied to larger datasets and hence allows for testing for—and even predicting—possible kinetic contributions of other *cis* and *trans*-acting frameshift stimulatory RNA elements. Furthermore, besides being a powerful tool to predict recoding efficiencies on a given frameshift site, our model should be able to distinguish between sequence patterns that allow efficient alternative frame translation in genomes. This way, in combination with bioinformatics, the free-energy model may also become a useful tool for further exploration of genomes for potential frameshift sites.

## Methods

### Translation assays

We used a variant of the original *dnaX* frameshifting site as described previously^20^. The mRNAs were prepared by in vitro transcription with T7 RNA polymerase^43,44^ and purified using the RNeasy midi kit (Qiagen) following the manufacturer’s recommendations. All mRNAs had the same length and contained the native stem-loop structure, but differed in the slippery site sequence, as shown in Supplementary Table 1. Ribosomes from *E. coli* MRE 600, EF-Tu, EF-G, and initiation factors, were prepared according to detailed protocols^37,45–48^. fMet-tRNA^fMet^ Phe-tRNA^Phe^, Val-tRNA^Val^, Ile-tRNA^Ile^, Glu-tRNA^Glu^, Lys-tRNA^Lys^, and Leu-tRNA^Leu^(CUN) were prepared as described previously^20,49^. Ala-tRNA^Ala^ and Gln-tRNA^Gln^ were prepared by hydrophobic tagging^49^. Aminoacyl-tRNAs (aa-tRNAs) were precipitated with ethanol and dissolved in water. Concentrations of aa-tRNAs were determined photometrically by absorbance measurements at 260 nm and by liquid–liquid scintillation counting where applicable (Ultima Gold XR, Perkin Elmer).

The experiments were carried out in buffer A (50 mM Tris–HCl [pH 7.5], 70 mM NH_4_Cl, 30 mM KCl, 7 mM MgCl_2_) supplemented with GTP (1 mM) at 37 °C. To prepare initiation complexes, 70S ribosomes (1 μM) were incubated with a 3−5-fold excess of mRNA, a 1.5-fold excess of f[^3^H]Met-tRNA^fMet^ and a 1.2-fold excess of IF1, IF2, and IF3 each in buffer A for 30 min. Initiation complexes were purified by centrifugation through a sucrose cushion (1.1 M) in buffer A. Ternary complexes were prepared by incubating EF-Tu (two-fold excess over aa-tRNA) together with GTP (1 mM), phosphoenolpyruvate (3 mM), and pyruvate kinase (0.1 mg/ml) in buffer A for 15 min and then with the purified aa-tRNAs for 1 min. Translation experiments were performed in buffer A at 37 °C either as end-point experiments (60–120 s incubation) by hand or using a KinTek RQF3 quench-flow apparatus. Translation experiments were carried out by rapidly mixing initiation complexes (0.2 μM after mixing) with the respective ternary complexes as indicated (1 μM) and EF-G (2 μM) with GTP (1 mM). Reactions were quenched by the addition of KOH (0.5 M), and peptides were released by incubation for 30 min at 37 °C. After neutralization with acetic acid, samples were analyzed by HPLC (LiChroSpher100 RP-8 HPLC column, Merck) using a gradient of acetonitrile in 0.1% heptafluorobutyric acid (HFBA) in order to separate the basic (MAK and MAKK) peptides or in 0.1% trifluoroacetic acid. The elution times of the reaction products were established using a set of model peptides synthesized in vitro, in which one of the amino acids is [^14^C]-labeled^20^. The extent of product formation was determined from the ratio of f[^3^H]Met in the respective peak to the total ^3^H-radioactivity in the eluate. Quantification of f[^3^H]MetAlaLys[^14^C]GluVal was based on the amount of [^14^C]Glu radioactivity in peptides. FS was calculated from the end points of in vitro translation experiments; the values are mean ± s.d. (*n* = 3 or more independent experiments). Time courses were evaluated by numerical integration using MatLab software according to the model shown in Supplementary Fig. 1c. An analogous model was used to evaluate peptide synthesis in the presence and absence of Glu-tRNA^Glu^. The fraction of non-progressing ribosomes was taken into account by drop-off parameters in the model. Standard deviations of rates were determined by numerical integration using in-built software routines assuming 95% confidence limit. All kinetic experiments were repeated at least twice.

### FS as a function of base-pair free-energy differences

For the −1 frameshifting of the *dnaX* mRNA, a stem loop downstream of the slippery sequence is essential^26^. The stem loop has been proposed to pause the ribosome, thereby increasing the time the tRNAs interact with the slippery sequence codons^26^. If the pausing time is long enough, frameshifting efficiency FS, i.e., the probability of ending up in the −1-frame, is only determined by the free-energy difference between the 0-frame and −1-frame. To test if the free-energy differences suffice to explain the measured FS values, we introduce a free-energy model of frameshifting.

Given are frameshift efficiencies ***FS***_experiment_ = (*FS*_experiment,1_,⋯, *FS*_experiment,*N*_) measured for *N* different mRNA sequences (Fig. 1, Supplementary Table 1). Here, we analyze *N* = 64 different mRNA variants.

Upon frameshifting, the mRNA–tRNA base-pairing nucleotides change and therefore the free energy of the mRNA–tRNA interaction changes. Let us assume that the efficiency FS_*i*_ of the *i*th mRNA variant solely depends on the difference between the free energy of the −1-frame *G*_−1,*i*_ and of 0-frame *G*_0,*i*_. In this case, the efficiencies can be determined by the ratio of Boltzmann probabilities

1 $${\mathrm{{FS}}}_i = \frac{{{\mathrm{{exp}}}\left( { - \frac{{\Delta G_i}}{{k_{\mathrm{{B}}}T}}} \right)}}{{1 + {\mathrm{{exp}}}\left( { - \frac{{\Delta G_i}}{{k_{\mathrm{{B}}}T}}} \right)}},\Delta G_i = G_{ - 1,i} - G_{0,i}\, \Longrightarrow \, \Delta G_i = - k_{\mathrm{{B}}}T\,{\mathrm{ln}}\left( {\frac{{{\mathrm{{FS}}}_i}}{{1 - {\mathrm{{FS}}}_i}}} \right).$$

Hence, if Δ*G*_*i*_ for the *i*th mRNA variant is negative, i.e., the free energy of the −1-frame is lower than that of the 0-frame, the corresponding FS_*i*_ is larger than 50%.

During *dnaX* −1 frameshifting, the two tRNAs, which were located in the ribosomal P and A sites prior to translocation, are interacting with the first and second codon of the mRNA, respectively. Due to the shift of the reading frame, the base pairs in the codons may change, depending on the sequence (compare Fig. 1a). Each codon consists of three base pairs which are denoted by either P1, P2, P3 or A1, A2, A3, where the letter corresponds to the P-site or A-site codon and the number corresponds to the position of the base pair in the codon.

The second assumption, which will be tested using the free-energy model, is that the free-energy difference Δ*G*_*i*_ only arises from the free-energy differences of the base pairs at the individual positions present in the 0-frame and −1-frame, and further, that these are additive. Base-pair changes are denoted by their codon position and the two base pairs, e.g., P1 A·U → C·U corresponds to changing the mRNA–tRNA base pair A·U into a C·U base pair in the first position of the first codon. In the *N* = 64 mRNA sequences, *n* = 16 base-pair changes are found: at the first positions of both codons (P1 and A1), six base-pair changes each (A·U → C·U, A·U → U·U, A·U → G·U, U·A → A·A, U·A → C·A, U·A → G·A) and at the third positions (P3 and A3), two base-pair changes each (G·S → A·S, C·G → U·G). The base pairs at the second positions (P2 and A2) remain unchanged upon frameshifting for all the 64 mRNA variants and therefore do not contribute to the free-energy differences Δ*G*_*i*_. Here, S denotes the modified nucleotide mnm^5^s^2^U, which is present in the anticodon of tRNA^Lys^ (Supplementary Fig. 4).

The estimated free-energy difference Δ*G*_est,*i*_ between the 0-frame and the −1-frame for the ***i***th mRNA sequence can then be calculated from the sum of the individual free-energy differences Δ*G*_bp,*j*_ of the base-pair combinations

2 $$\Delta G_{{\mathrm{est}},i} = \mathop {\sum}\limits_j^n {M_{i,j}} \Delta G_{{\mathrm{bp}},j},$$

where *M*_*i*,*j*_ is 1 if the free-energy difference Δ*G*_bp,*j*_ contributes to Δ*G*_*i*_, −1 if −Δ*G*_bp,*j*_ contributes to Δ*G*_*i*_, and 0 if Δ*G*_bp,*j*_ does not contribute to Δ*G*_*i*_. For a compact notation, the base-pair free-energy differences are combined into a vector

$$\begin{array}{*{20}{l}} {{\mathrm{\Delta }}{\mathbf{G}}_{{\mathrm{bp}}}} \hfill & = \hfill & {\left( {\Delta G_{{\mathrm{bp}},1},\Delta G_{{\mathrm{bp}},2}, \cdots ,\Delta G_{{\mathrm{bp}},n}} \right)^{\top} } \hfill \\ {} \hfill & = \hfill & {\left( {\Delta G\left( {{\mathrm{P}}1\,{\mathrm{A}} \cdot {\mathrm{U}} \to {\mathrm{C}} \cdot {\mathrm{U}}} \right),\Delta G\left( {{\mathrm{P}}1\,{\mathrm{A}} \cdot {\mathrm{U}} \to {\mathrm{U}} \cdot {\mathrm{U}}} \right), \cdots ,\Delta G\left( {{\mathrm{A}}3\,{\mathrm{C}} \cdot {\mathrm{G}} \to {\mathrm{U}} \cdot {\mathrm{G}}} \right)} \right)^\top .} \hfill \end{array}$$

Writing all the estimated free-energy differences Δ*G*_est,*i*_ of the *N* mRNA sequences as a vector,

3 $${\mathbf{\Delta}} {\mathbf{G}}_{{\mathrm{est}}} = \left( {{\mathrm{\Delta }}G_{{\mathrm{est}},1},{\mathrm{\Delta }}G_2, \cdots ,{\mathrm{\Delta }}G_N} \right)^\top,$$

Equation (2) can be expressed as a matrix multiplication, **ΔG**_est_ = **M** · **ΔG**_bp_, where **M** is an *N* × *n* matrix with entries *M*_*i*,*j*_. From the estimated free-energy differences **ΔG**_est_, using Eq. (1), we can calculate the frameshift efficiencies **FS**_model_, which are now a function of the base-pair free-energy differences.

### Metropolis with Bayesian Inference

The aim is to find the individual base-pair free-energy differences **ΔG**_bp_ that best reproduce the measured frameshift efficiencies ***FS***_experiment_. Using Bayesian inference, the probability for the base-pair free-energies is

4 $$P({\mathbf{\Delta}} {\mathbf{G}}_{{\mathrm{bp}}}|{{\boldsymbol{FS}}_{\mathrm{{{experiment}}}}}) \propto P({{\boldsymbol{FS}}_{\mathrm{{{experiment}}}}}|{\mathbf{\Delta}} {\mathbf{G}}_{{\mathrm{bp}}}) \cdot P({\mathbf{\Delta}} {\mathbf{G}}_{{\mathrm{bp}}}),$$

where *P*(**ΔG**_bp_) is the prior probability of the base-pair free-energy differences, and *P*(***FS***_experiment_|**ΔG**_bp_) is the probability of observing specific frameshift efficiencies ***FS***_experiment_ for given base-pair free-energy differences **ΔG**_bp_,

5 $$P({\boldsymbol{FS}}_{{\mathrm{experiment}}}|{\mathbf{\Delta}} {\mathbf{G}}_{{\mathrm{bp}}}) = \mathop {\prod}\limits_i^N {\frac{1}{{\sqrt {2\pi \sigma _{{\mathrm{experiment}},i}^2} }}}\ {\mathrm{exp}}\left( { - \frac{{\left( {{\mathrm{{FS}}}_{{\mathrm{experiment}},i} - {\mathrm{{FS}}}_{{\mathrm{model}},i}} \right)^2}}{{2\sigma _{{\mathrm{experiment}},i}^2}}} \right),$$

where *σ*_experiment,*i*_ is the standard deviation of FS_experiment,*i*_ obtained from repeated measurements (Fig. 1b, Supplementary Table 1), and FS_model,*i*_ is the ***i***th entry of the vector of frameshift efficiencies ***FS***_model_ estimated from **ΔG**_bp_, using Eqs. (1) and (2).

The prior distribution *P*(**ΔG**_bp_) of the vector of base-pair free-energy differences **ΔG**_bp_ is

6 $$P({\mathbf{\Delta}} {\mathbf{G}}_{{\mathrm{bp}}}) = \mathop {\prod}\limits_j^n P (\Delta G_{{\mathrm{bp}},j}),$$

where *P*(Δ*G*_bp,*j*_) is the prior distribution of free-energy difference of the *j*th base pair. This prior distribution was chosen to be a uniform distribution between **−**25 and 25 kJ/mol.

*P*(**ΔG**_bp_|***FS***_experiment_) was sampled using the Metropolis Monte Carlo algorithm^50^ in two independent calculations with 10^6^ steps. To that aim, we used the function

7 $$f({\boldsymbol{FS}}_{{\mathrm{experiment}}},{\mathbf{\Delta}} {\mathbf{G}}_{{\mathrm{bp}}}) = P({\boldsymbol{FS}}_{{\mathrm{experiment}}}|{\mathbf{\Delta}} {\mathbf{G}}_{{\mathrm{bp}}}) \cdot P({\mathbf{\Delta}} {\mathbf{G}}_{{\mathrm{bp}}}),$$

which is proportional to the desired probability distribution (compare to Eq. (4)).

The initial free-energy difference values Δ*G*_bp,*j*_ were set to 0 kJ/mol and the function *f* was evaluated. For each metropolis step, *n* sub-steps were carried out. For each substep *j*, first, a new value for the Δ*G*_bp,*j*_ was drawn from a normal distribution centered on the current value with a *σ* of 0.2 kJ/mol. Then, the function *f* was evaluated with the new Δ*G*_bp,*j*_, and the ratio *α* of the new and previous value of *f* was used as the acceptance ratio: If *α* > 1, the new Δ*G*_bp,*j*_ was accepted. If *α* < 1, a random number *u* between 0 and 1 was drawn and the new Δ*G*_bp,*j*_ value was accepted if *u* ≤ *α* and rejected otherwise.

### Determination of independent free-energy differences

The mean *μ* and standard deviation *σ* of the probability distributions of the base-pair free-energy differences Δ*G*_bp,*j*_, obtained from Metropolis sampling of *P*(**ΔG**_bp_|***FS***_experiment_), are shown in Supplementary Fig. 5a. For 10 of the 16 base-pair combinations *σ* is small (Supplementary Fig. 5a, green background) showing that the Δ*G*_bp_ of these combinations is well determined.

For the remaining six base-pair combinations with large *σ* values, the absolute Δ*G*_bp_ values are not determined, but their Δ*G*_bp_ values sampled during the calculations show strong mutual correlations (Supplementary Fig. 6a). The Δ*G*_bp_ values of the three base-pair changes P3 G·S → A·S, A1 A·U → G·U, and A1 U·A → G·A show strong positive pairwise correlations. The same is observed for the other three base-pair changes P3 C·G **→** U·G, A1 A·U → C·U, and A1 U·A **→** C·A. Since strong positive correlations for pairs of Δ*G*_bp_ values means that the difference between them is determined, we ran additional Metropolis sampling calculations, now with Δ*G*_bp_ set to zero for P3 G·S → A·S (Supplementary Fig. 5b). As expected from the correlation, the *σ* of the Δ*G*_bp_ values for A1 A·U → G·U and A1 U·A → G·A is reduced. Analogously, setting Δ*G*_bp_ of P3 C·G → U·G to zero, leads to small *σ* values for A1 A·U → C·U and A1 U·A → C·A (Supplementary Fig. 5c). Setting both Δ*G*_bp_ values to zero at the same time, results in low *σ* values for all base-pair combinations (Supplementary Fig. 5d). This result enabled us to determine probability distribution for Δ*G*_bp_ values for pairs of base-pair changes (Fig. 2c).

To check the consistency of the free-energy model, we recalculated the FE values, using Eqs. (1) and (2), from all the Δ*G*_bp_ values after omitting the first 20% of the Metropolis steps. The rmsd between the measured ***FE***_experiment_ values and those obtained from the model ***FE***_model_ was 2.52% for all of these cases (Supplementary Fig. 5a–d, Fig. 2d). To further test whether the ***FE***_model_ values have converged, we first extracted intervals of the sampled Δ*G*_bp_ values from the first *N*_steps_ Metropolis steps (Supplementary Fig. 7a, number of steps). For each interval the first 20% Metropolis steps were omitted and then, the rmsd between the ***FE***_experiment_ values and the mean ***FE***_model_ values obtained from the Δ*G*_bp_ values was calculated. For the two independent calculations, the rmsd drops to the same value showing that the FE values have converged (Supplementary Fig. 7a).

For cross validation, iteratively, each mRNA variant was selected and the distributions of Δ*G*_bp_ were calculated from the FE values of all other mRNA variants. Next, the FE for the selected mRNA variant was predicted from the obtained Δ*G*_bp_ values (Fig. 2e). The rmsd between the predicted and the measured FE values as a function of Metropolis steps is shown in Supplementary Fig. 7b and converges to 4.1%.

### Estimation of the kinetic contribution to frameshifting

So far, the frameshifting efficiency was assumed to be solely determined by the free-energy difference between the 0-frame and the −1-frame Δ*G*. Next, to challenge this assumption and to test if the frameshifting efficiency also depends on kinetics, we expanded the model by a term that describes kinetic effects. Given the two states, 0-frame and −1-frame, and the rates between these states, *k*_0,−1_ and *k*_−1,0_, the master equation for the time-dependent probability of being in the −1-frame *P*_−1_(*t*) can be written as

8 $$\frac{{{\mathrm{{d}}}P_{ - 1}(t)}}{{{\mathrm{{d}}}t}} = k_{0, - 1}\left[ {1 - P_{ - 1}(t)} \right] - k_{ - 1,0}P_{ - 1}(t).$$

Assuming that the system starts in the 0-frame and, hence, *P*_−1_(0) = 0, the solution of the master equation is given by

9 $$P_{ - 1}(t) = \frac{{k_{0, - 1}}}{{k_{0, - 1} + k_{ - 1,0}}}\left[ {1 - {\mathrm{{exp}}}( - (k_{0, - 1} + k_{ - 1,0})t)} \right].$$

With transition rates given by Arrhenius’s law

10 $$k_{0, - 1} = A\, {\mathrm{{exp}}}\left( { - \frac{{\Delta G^{\mathrm{\ddagger }}}}{{k_{\mathrm{{B}}}T}}} \right),\quad k_{ - 1,0} = A\, {\mathrm{{exp}}}\left( { - \frac{{\Delta G^{\mathrm{\ddagger }} - \Delta G}}{{k_{\mathrm{{B}}}T}}} \right),$$

with barrier height $\Delta G^{\mathrm{\ddagger }}$ and attempt frequency *A*, the probability of being in the −1-frame reads

11 $$P_{ - 1}(t) = {\mathrm{{FS}}}\left[ {1 - {\mathrm{exp}}( - (k_{0, - 1} + k_{ - 1,0})t)} \right],$$

where FS is the equilibrium frameshift efficiency as described in Eq. (1).

Obviously, for *t* → ∞, the probability *P*_−1_(*t*) converges to *P*_−1_(*t* → ∞) = *FS*, the equilibrium efficiency. The time window for transitions between the two frames is limited and ends with the completion of tRNA translocation. We define the kinetic factor *κ* = exp(−(*k*_0,−1_ + *k*_−1,0_)*τ*) with *τ* as the length of this time window. Therefore the frameshifting efficiency *FS*_kinetic_, obtained from the model including kinetic effects, is then *FS*_kinetic_ = *P*_−1_(*τ*) = *FS*(1−*κ*). Substituting *k*_0,−1_ and *k*_0,−1_ using Eq. (10) results in

12 $$\kappa = {\mathrm{{exp}}}\left( { - A\tau \, {\mathrm{exp}}\left( { - \frac{{\Delta G^{\mathrm{\ddagger }}}}{{k_{\mathrm{{B}}}T}}} \right)\left[ {1 + {\mathrm{exp}}\left( {\frac{{\Delta G}}{{k_{\mathrm{{B}}}T}}} \right)} \right]} \right).$$

This model of the possible non-equilibrium dynamics of frameshifting leaves

13 $$C = A \tau \, {\mathrm{exp}}\left( { - \frac{{{\mathrm{\Delta }}G^{\mathrm{\ddagger }}}}{{k_BT}}} \right)$$

as the only remaining unknown parameter. The kinetic factor *κ* is different for different mRNA variants, depending on the Δ*G* which in turn depends on the base-pair free-energy differences.

Since we are interested in the kinetic contribution to frameshifting, we performed additional Metropolis sampling calculations, with the mean $\bar \kappa$ of all kinetic factors included as a nuisance parameter. The probability for the base-pair free-energies is then

14 $$P({\mathbf{\Delta}} {\mathbf{G}}_{{\mathrm{bp}}}|{\boldsymbol{FS}}_{{\mathrm{experiment}}}) \propto P({\boldsymbol{FS}}_{{\mathrm{experiment}}}|{\mathbf{\Delta}} {\mathbf{G}}_{{\mathrm{bp}}}) \cdot P({\mathbf{\Delta}} {\mathbf{G}}_{{\mathrm{bp}}}) \cdot P(\bar \kappa ),$$

where $P(\bar \kappa )$ is the prior distribution of $\bar \kappa$ for which a uniform distribution between 0 and 1 was chosen. To sample the probability, in each Metropolis step, the parameter *C* which results in the mean kinetic factor $\bar \kappa$ was calculated and from that the $\kappa$ value for each sequence.

Two independent calculations were carried out with 10^6^ Metropolis steps each, as described above, now with an additional sub step for drawing a new value for $\bar \kappa$ from a normal distribution centered on the current value of $\bar \kappa$ with $\sigma = 0.001$. The obtained probability distribution of $\bar \kappa$ is shown in Fig. 3b.

### Independent set of FS values

To further test the validity of the free-energy model, we applied it to an independent data set of 21 FS values previously published by Tsuchihashi et al.^26^ (Supplementary Table 3). The codon–anticodon base-pair interactions of the 21 mRNA variants, can be described by 26 free-energy differences (Table 1).

**Table 1:** Base-pair changes contributing to the codon–anticodon interactions in the mRNA sequences of the Tsuchihashi data set^26^

Base-pair position	Base-pair change
P1	**A·U** → **C·U**, **A·U**→**U·U**, **A·U** → **G·U**, G·C→A·C
P2	A·U → G·U, U·A→A·A, G·C→A·C, C·G→A·G
P3	**G·S** → **A·S**, A·}→U·}, A·{→G·{, A·V→C·V, C·Q→A·Q, U·Q→A·Q, A·)→U·)
A1	**A·U** → **U·U**, **A·U** → **G·U**, **A·U** → **C·U**, G·C→A·C
A2	A·U → G·U, U·A→A·A
A3	**G·S** → **A·S**, C·Q→A·Q, G·M→U·M, U·Q→A·Q, A·{→G·{

Modified nucleotides: S = mnm^5^s^2^U, {=mnm^5^U,) = cmnm^5^U, Q = queuosine, V = cmo^5^U, M = ac^4^C,} = 2-lysidine

Changes highlighted in bold are present in our data set as well (compare Fig. 2a–c)

Metropolis sampling of *P*(**ΔG**_bp_|***FS***_experiment_) was carried out as described above (without kinetic factor), now for the 26 free-energy differences and 21 FS values. In the first step (Supplementary Fig. 8a), the Δ*G*_bp_ of 9 base-pair changes have a low standard deviation *σ*, showing that their free-energy differences are determined by the Tsuchihashi FS data set (green histograms in Figs. 2a, b, and 3a, b). In steps 2–9 (Supplementary Fig. 8b–i), iteratively, the Δ*G*_bp_ of a base-pair change that had a large *σ* in all previous steps was set to 0 kJ/mol. Monitoring for which base-pair changes, the *σ* values were reduced, we could identify which Δ*G*_bp_ values of pairs of base-pair changes were determined (Figs. 2c and 3c). Finally, in step 10, all the Δ*G*_bp_ values set to 0 kJ/mol in steps 2–9 were set to 0 kJ/mol at the same time (Supplementary Fig. 8j). For all steps 1–10, the rmsd for the measured FS values and those obtained from the model was found to be between 2.51% and 2.59%. For the Metropolis calculations of step 10 (Supplementary Fig. 8j), the rmsd was calculated as a function of Metropolis steps (Supplementary Fig. 7c) showing the convergence of the Metropolis sampling.

### Comparison of P-site and A-site base pairs

The environment of the base pairs is different in the ribosomal P and A sites. The free-energy differences for C·A → C·U and G·A → G·U could not be obtained directly from the set of FS values. Under the assumption that A·U and U·A base pairs have the same free energy, the free-energy difference for P1 C·A → C·U was obtained by subtracting Δ*G*_bp_ of P1 U·A → C·A from that of A·U → C·U (Fig. 2a, fifth and first rows). For the corresponding A-site free-energy difference, the Δ*G*_bp_ of a pair of base-pair changes P3 C·G → U·G and A1 U·A → C·A (Fig. 2c, fourth row) was subtracted from that of another pair P3 C·G → U·G and A1 A·U → C·U (Fig. 2c, third row). For G·A → G·U, we used the Δ*G*_bp_ of Fig. 2a, third and sixth rows, for the P site, and Fig. 2c, first and second rows, for the A site.

### Reporting summary

Further information on research design is available in the Nature Research Reporting Summary linked to this article.

## Supplementary information

Supplementary Information

Peer Review

Reporting Summary

## Data Availability

The authors declare that the data supporting the findings of this study are available within the paper and its supplementary information files.

## Code availability

The code used for Monte Carlo sampling and analysis is available from the corresponding author upon request.
