# Supplementary material for: Thermodynamic control of −1 programmed ribosomal frameshifting
Source: Nat Commun. 2019 Oct 10;10:4598. doi: 10.1038/s41467-019-12648-x (PMC6787027; doi:10.1038/s41467-019-12648-x)
Supplement: Supplementary file 1 — Supplementary Information [file 41467_2019_12648_MOESM1_ESM.pdf]

Thermodynamic Control of –1 Programmed Ribosomal Frameshifting
Suppementary Information

Lars V. Bock[1,5], Neva Caliskan[2,3,5], Natalia Korniy[4], Frank Peske[4], Marina V. Rodnina[4], and Helmut Grubmüller*[1]

[1]Theoretical and Computational Biophysics, Max Planck Institute for Biophysical Chemistry, 37077 Göttingen , Germany.
[2]Helmholtz Institute for RNA-based Infection Research (HIRI), Helmholtz Centre for Infection Research (HZI), 97080 Würzburg, Germany
[3]Medical Faculty, University of Würzburg, 97080 Würzburg, Germany
[4]Department of Physical Biochemistry, Max Planck Institute for Biophysical Chemistry, 37077 Göttingen, Germany
[5]These authors contributed equally to this work

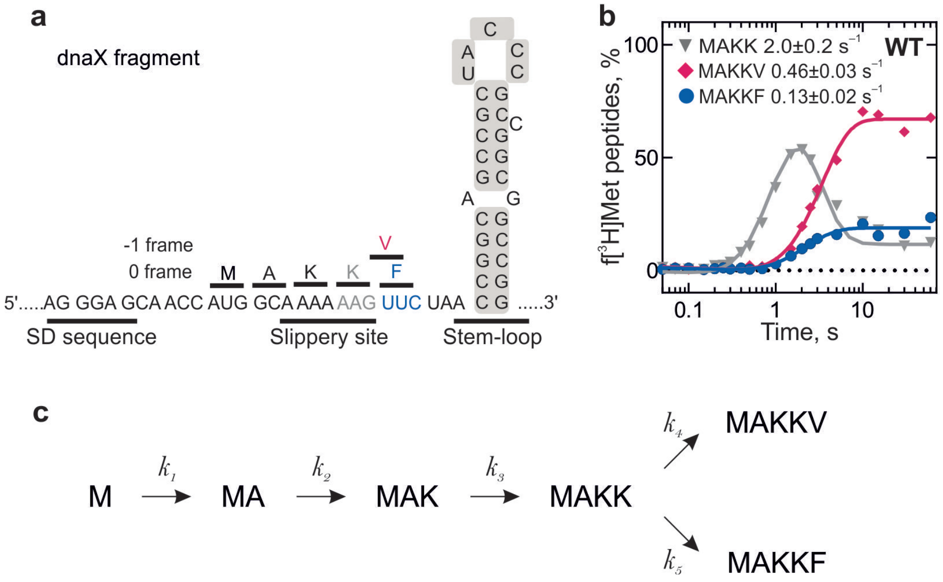

Supplementary Fig. 1 *dnaX* frameshifting region used in this study. (**a**) Wild type *dnaX* slippery sequence optimized for *in vitro* translation in the reconstituted *in vitro* translation system from *E. coli*[1]. Upon –1PRF the identity of the codons following the AAA AAG slippery site changes from 0-frame UUC (Phe) to –1 frame GUU (Val). (**b**) Time courses of amino acid incorporation upon translation of the mRNA shown in (a). Peptides are MAKK (gray), MAKKV (red), and MAKKF (blue). Numbers represent rate constants of amino acid incorporation, as determined by global fitting (Table 2M from[1]) according to the model shown in (**c**). Global fits are shown as continuous lines.

*Corresponding author: hgrubmu@gwdg.de

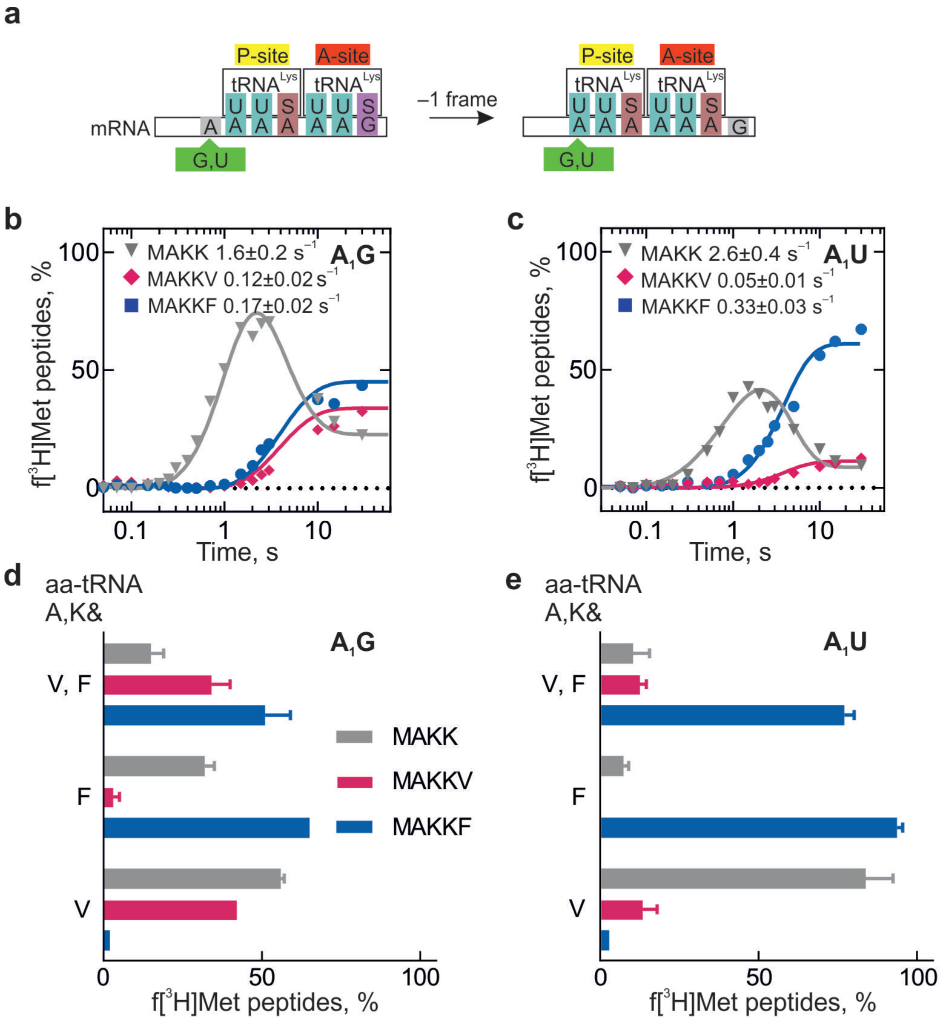

Supplementary Fig. 2 Frameshifting on *dnaX* variants with mutations A_1G and A_1U. (**a**) Schematic of wt and mutated sequences. (**b**–**c**) Time courses of amino acid incorporation upon translation of *dnaX* A_1G (**b**) and A_1U (**c**) mRNA variants. Peptides are MAKK (gray), MAKKV (red), and MAKKF (blue). Numbers represent rate constants of amino acid incorporation, as determined by global fitting (Table 2). Global fits are shown as continuous lines. (**d**–**e**) Efficiency of amino acid incorporation in –1- and 0-frame on A_1G (**d**) and A_1U (**e**) mRNA monitored at the end point of translation (60–120 s) in the presence and absence of Val-tRNA^{Val} (V) and Phe-tRNA^{Phe} (F) decoding the –1 and 0 frames, respectively. Ala-tRNA^{Ala} (A) and Lys-tRNA^{Lys} (K) were present in all experiments. The efficiency values are mean ± s.d. from independent experiments (N≥3).

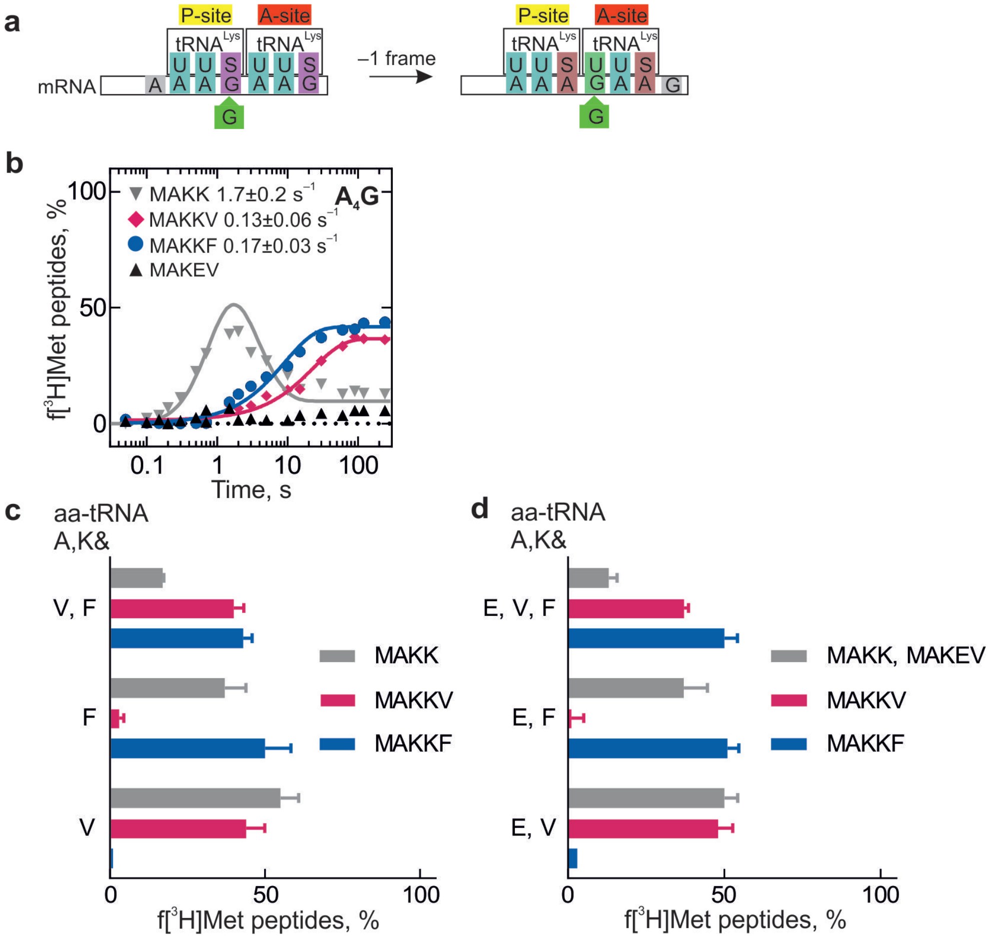

Supplementary Fig. 3 Translation of the slippery-motif mutant A_4G. (**a**) Schematic of wt and mutated sequences. (**b**) Codon walk over the *dnaX* A_4G mRNA variant. Time courses of peptide synthesis are monitored with ribosomes programmed with A_4G mRNA in the presence of EF-Tu, aa-tRNAs for A, K, V, F and E and EF-G. Peptides are MAKK (gray), MAKKV (red), MAKKF (blue), and MAKEV (black). Numbers represent rate constants of amino-acid incorporation, as determined by global fitting (Table 2). Global fits are shown as continuous lines. (**c**) Efficiency of amino-acid incorporation in –1 and 0 frame on A_4G complexes monitored at the end of translation (60–120 s) in the presence and absence of Val-tRNA^{Val} (V) and Phe-tRNA^{Phe} (F), decoding the –1 and 0 frames, respectively. Ala-tRNA^{Ala} (A) and Lys-tRNA^{Lys} (K) were present in all experiments. (**d**) same as (**c**), in the presence and absence of Glu-tRNA^{Glu} (E). The efficiency values are mean ± s.d. from independent experiments (N≥3).

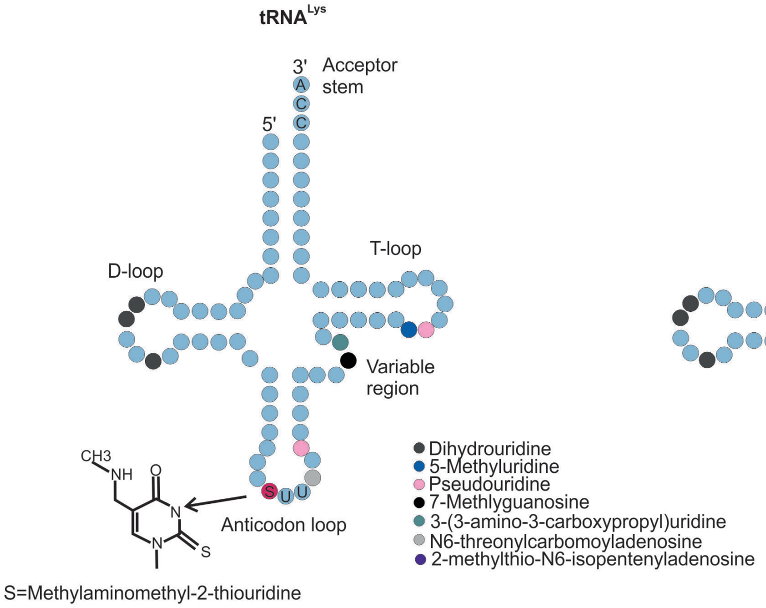

Supplementary Fig. 4 Comparison of the $tRNA^{Lys}$ and $tRNA^{Phe}$ and the modified bases of the two tRNAs.

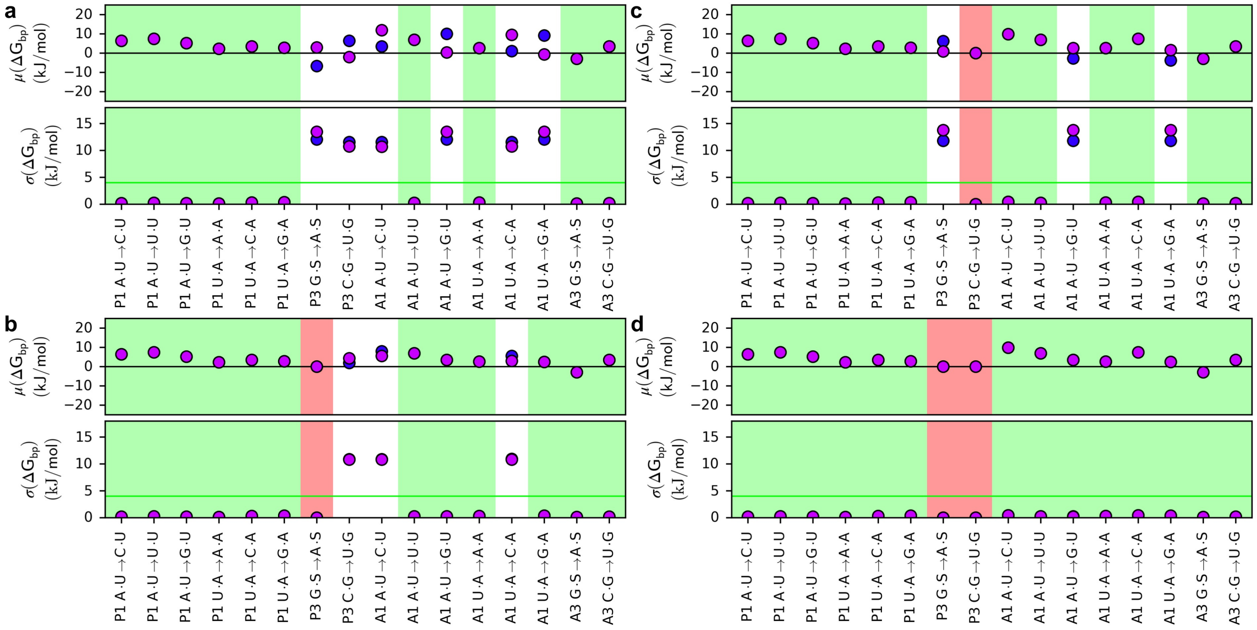

Supplementary Fig. 5 Determination of independent free-energy differences from the presented set of FS values. (**a**) Step 1: For each combination of base pairs, the mean μ and standard deviation σ of the free-energy difference ΔG_{bp} obtained from two independent Metropolis sampling calculations (blue and pink cirlces) is shown. Base-pair combinations for which σ is below the threshold of 4 kJ/mol (green horizontal line) are highlighted with green background. (**b**, **c**, **d**) Steps 2, 3, and 4: base-pair combinations for which ΔG_{bp} was set to 0 kJ/mol are highlighted with red background.

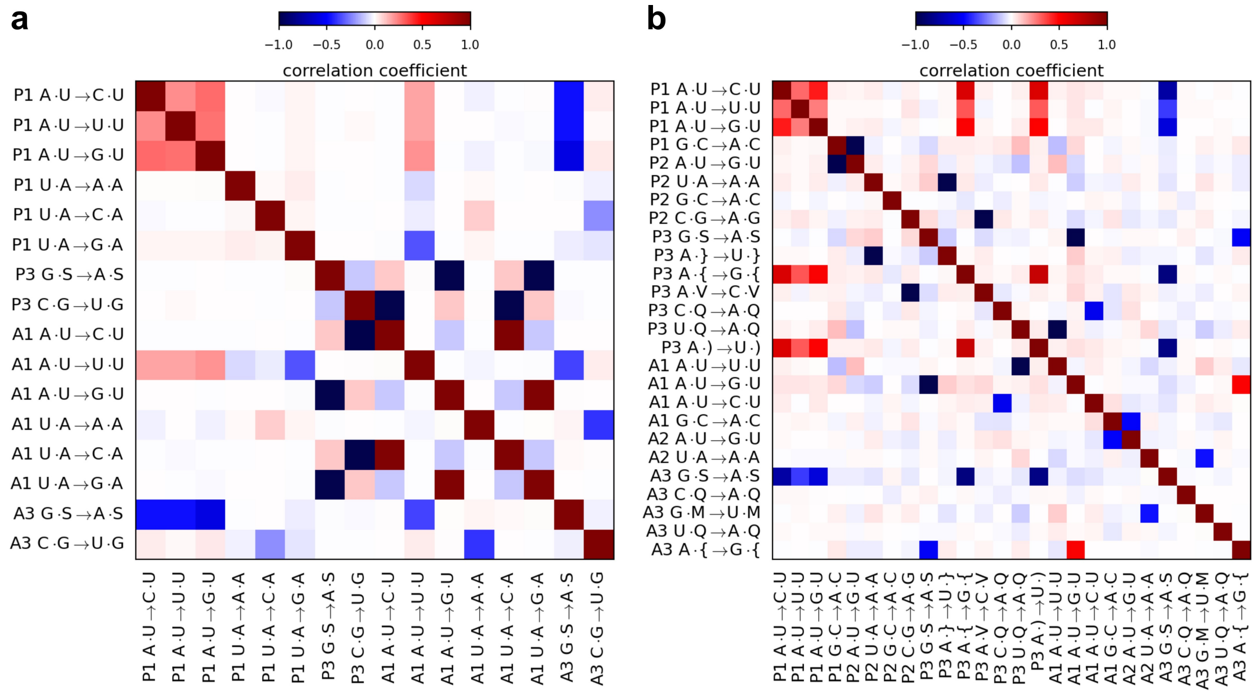

Supplementary Fig. 6 Correlation of free-energy differences. The mean correlation coefficient of the $\Delta G_{\rm bp}$ values for all pairs of base-pair combinations sampled in the two independent Metropolis calculations of the full data set (Fig. 1b) (**a**) and of the Tsuchihashi data set [2] (**b**).

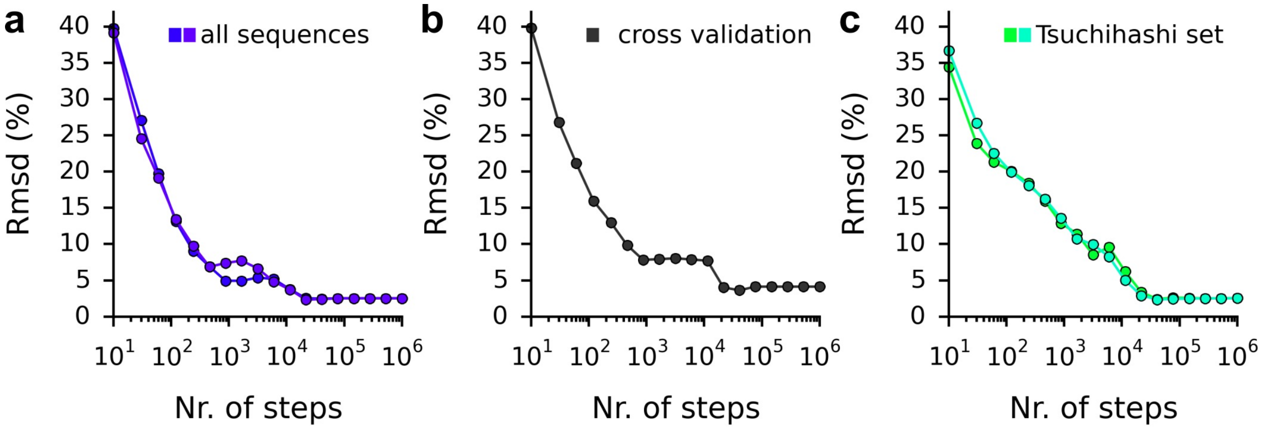

Supplementary Fig. 7 Convergence of Metropolis calculations. The root mean square deviation (rmsd) between the FS values from experiment and from the model are shown as a function of Metropolis steps. (**a**) Rmsd for two independent calculations including all 64 FS values (Fig. 1b). (**b**) Rmsd for all 64 FS values, each predicted, iteratively, from all other FS values for cross validation. (**a**) Rmsd for two independent calculations including 21 FS values from the Tsuchihashi data set [2].

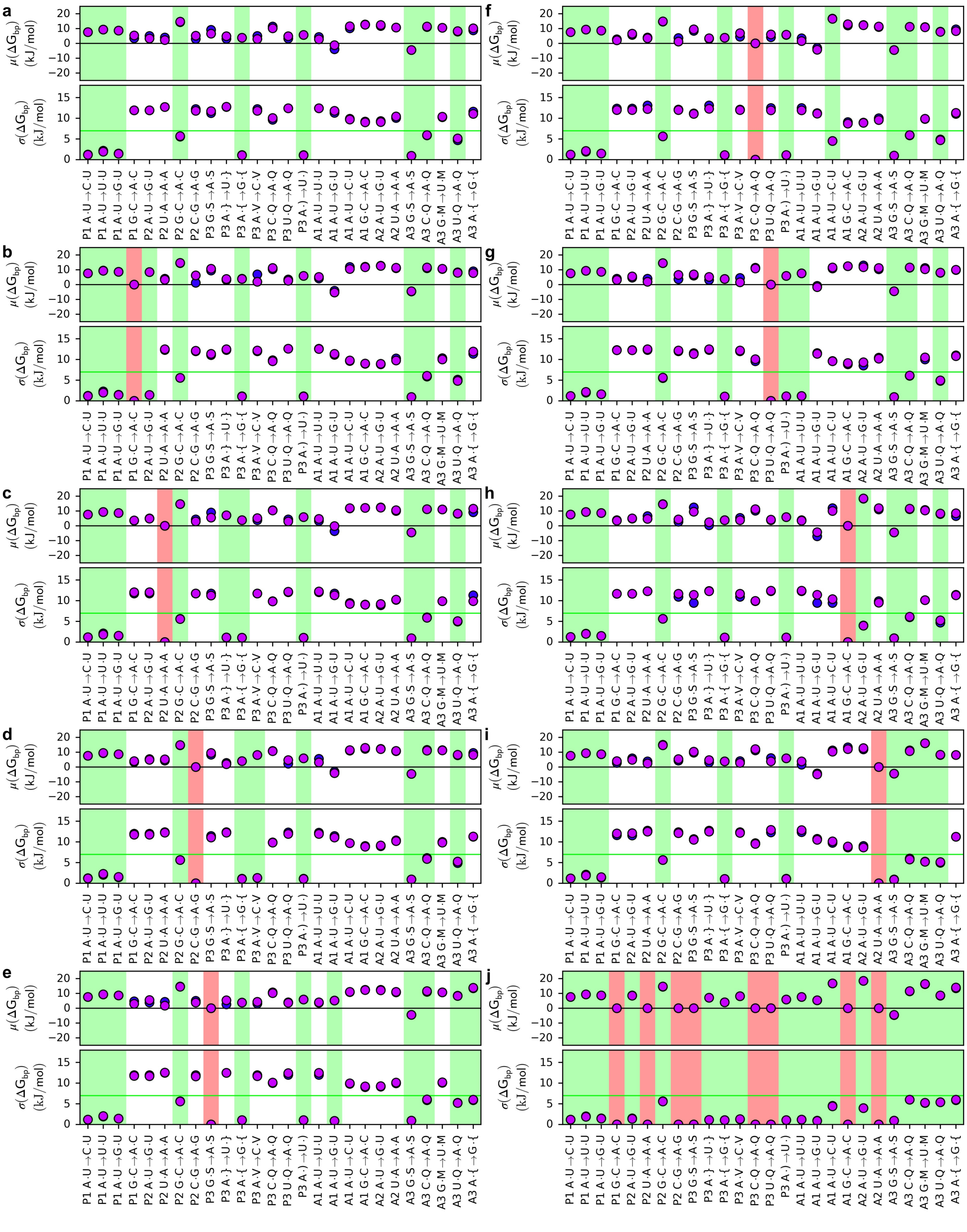

Supplementary Fig. 8 Determination of independent free-energy differences from the Tsuchihashi set of FS values. (**a**) Step 1: For each combination of base pairs, the mean μ and standard deviation σ of the free-energy difference ΔG_{bp} obtained from two independent Metropolis sampling calculations (blue and pink cirlces) is shown. Base-pair combinations for which σ is below the threshold of 8 kJ/mol (green horizontal line) are highlighted with green background. (**b**–**j**) Steps 2–10: Base-pair combinations for which ΔG_{bp} was set to 0 kJ/mol are highlighted with red background.

Supplementary Table 1 Measured FS for slippery sequence variants encoding for four pairs of amino acids in the 0-frame. The base pairs between the mRNA codons (lower sequence) and the tRNA anticodons (upper sequence) are shown, along with the FS values of repeated measurements, mean values, and the standard devitations (std).

Lys - Lys				Phe - Phe				Lys - Phe				Phe - Lys			
0-frame base pairs	FS (%)	mean (%)	std (%)	0-frame base pairs	FS (%)	mean (%)	std (%)	0-frame base pairs	FS (%)	mean (%)	std (%)	0-frame base pairs	FS (%)	mean (%)	std (%)
UUS UUS<br>A AAA AAA	54,45,48, 44,49	48.0	3.5	AAG AAG<br>A UUU UUU	28,28,30	29.0	1.0	UUS AAG<br>A AAA UUU	20,28,19	22.7	4.5	AAG UUS<br>A UUU AAA	6,1,5	4.0	2.2
UUS UUS<br>A AAA AAG	79,79,79, 81,81	79.8	1.0	AAG AAG<br>A UUU UUC	5,12,8	8.3	2.9	UUS AAG<br>A AAA UUC	7,7,5	6.3	1.0	AAG UUS<br>A UUU AAG	8,3,5	5.3	2.1
UUS UUS<br>A AAG AAA	17,20,22	19.7	2.1	AAG AAG<br>A UUC UUU	3,5,6	4.7	1.2	UUS AAG<br>A AAG UUU	13,26,21	20.0	5.4	AAG UUS<br>A UUC AAA	4,1,5	3.3	1.7
UUS UUS<br>A AAG AAG	39,45,47	43.7	3.4	AAG AAG<br>A UUC UUC	2,2,1	1.7	1.0	UUS AAG<br>A AAG UUC	9,5,5	6.3	1.9	AAG UUS<br>A UUC AAG	5,8,8	7.0	1.4
UUS UUS<br>C AAA AAA	8,10,9	9.0	1.0	AAG AAG<br>C UUU UUU	22,24,18	21.3	2.5	UUS AAG<br>C AAA UUU	4,5,6	5.0	1.0	AAG UUS<br>C UUU AAA	5,2,7	4.7	2.1
UUS UUS<br>C AAA AAG	18,19,21	19.3	1.2	AAG AAG<br>C UUU UUC	5,6,7	6.0	1.0	UUS AAG<br>C AAA UUC	4,3,3	3.3	1.0	AAG UUS<br>C UUU AAG	3,3,9	5.0	2.8
UUS UUS<br>C AAG AAA	3,2,3, 2	2.5	1.0	AAG AAG<br>C UUC UUU	3,2,1	2.0	1.0	UUS AAG<br>C AAG UUU	5,6,3	4.7	1.2	AAG UUS<br>C UUC AAA	5,1,4	3.3	1.7
UUS UUS<br>C AAG AAG	7,10,7	8.0	1.4	AAG AAG<br>C UUC UUC	2,2,1	1.7	1.0	UUS AAG<br>C AAG UUC	3,5,3	3.7	1.0	AAG UUS<br>C UUC AAG	2,3,7	4.0	2.2
UUS UUS<br>U AAA AAA	4,6,7	5.7	1.2	AAG AAG<br>U UUU UUU	40,45,49	44.7	3.7	UUS AAG<br>U AAA UUU	5,7,4	5.3	1.2	AAG UUS<br>U UUU AAA	9,6,8	7.7	1.2
UUS UUS<br>U AAA AAG	13,13,15	13.7	1.0	AAG AAG<br>U UUU UUC	21,23,22	22.0	1.0	UUS AAG<br>U AAA UUC	2,2,1	1.7	1.0	AAG UUS<br>U UUU AAG	20,19,23	20.7	1.7
UUS UUS<br>U AAG AAA	3,3,4	3.3	1.0	AAG AAG<br>U UUC UUU	4,5,3	4.0	1.0	UUS AAG<br>U AAG UUU	3,2,5	3.3	1.2	AAG UUS<br>U UUC AAA	6,1,6	4.3	2.4
UUS UUS<br>U AAG AAG	4,7,7	6.0	1.4	AAG AAG<br>U UUC UUC	3,2,3	2.7	1.0	UUS AAG<br>U AAG UUC	1,2,1	1.3	1.0	AAG UUS<br>U UUC AAG	5,4,5	4.7	1.0
UUS UUS<br>G AAA AAA	13,10,15	12.7	2.1	AAG AAG<br>G UUU UUU	31,26,22	26.3	3.7	UUS AAG<br>G AAA UUU	11,8,10	9.7	1.2	AAG UUS<br>G UUU AAA	5,2,7	4.7	2.1
UUS UUS<br>G AAA AAG	27,28,30	28.3	1.2	AAG AAG<br>G UUU UUC	9,14,14	12.3	2.4	UUS AAG<br>G AAA UUC	2,6,4	4.0	1.6	AAG UUS<br>G UUU AAG	3,5,6	4.7	1.2
UUS UUS<br>G AAG AAA	5,7,6	6.0	1.0	AAG AAG<br>G UUC UUU	2,4,5	3.7	1.2	UUS AAG<br>G AAG UUU	6,6,7	6.3	1.0	AAG UUS<br>G UUC AAA	4,3,4	3.7	1.0
UUS UUS<br>G AAG AAG	7,10,11	9.3	1.7	AAG AAG<br>G UUC UUC	2,2,1	1.7	1.0	UUS AAG<br>G AAG UUC	7,6,4	5.7	1.2	AAG UUS<br>G UUC AAG	5,2,5	4.0	1.4

Supplementary Table 2 Summary of rate constants of elemental translation steps of MAKK (k_{Lys2}), MAKKV (k_{Val}) MAKKF (k_{Phe}) upon –1 frameshift on the model dnaX frameshift constructs were determined by global fitting of the data shown in Fig. 1b, Fig. 2d,e; Fig. 3d and in Caliskan et al.[1]. Errors are s.e.m. of the fit. QF, Quench flow. The frameshifting efficiency was calculated from end points of *in vitro* translation experiments shown in Fig. 1b, Fig. 2b,c and Fig. 3b; the values are mean ± s.d. (n=3 independent experiments). [a] previously published[1].

mRNA variant	Rates (s^{-1})			FS (%)	
	k_{Lys2}	k_{Val}	k_{Phe}	QF	Endpoint
A1G	1.6±0.2	0.12±0.03	0.17±0.02	41±2	34±6
A1U	2.6±0.3	0.05±0.01	0.33±0.03	19±2	12±2
A4G	1.8±0.3	0.13±0.01	0.17±0.02	43±1	38±2
WT (SS/SL)[a]	2.0±0.2	0.46±0.03	0.13±0.02	78±1	72±2
–/SL[a]	1.3±0.1	0.03±0.01	0.3±0.0	9±3	4±1
SS/–[a]	2.2±0.1	0.7±0.03	2.3±0.1	23±2	16±5
–/–[a]	2.2±0.5	0.03±0.01	2.9±0.6	1±0	0

Supplementary Table 3 FS values from Tsuchihashi et al.[2]. The 0-frame base pairs between the mRNA codons (lower sequence) and the tRNA anticodons (upper sequence) as used in the free-energy model are shown, along with the FS valus. In the model, a standard deviation of 5% was assumed. The nomenclature for nucleotide modifications was taken from the MODOMICS database[3]

0-frame base pairs	FS (%)	0-frame base pairs	FS (%)	0-frame base pairs	FS (%)	0-frame base pairs	FS (%)
UUS UUS<br>A AAA AAG	81	UUS UUS<br>G AAA AAG	20	CUS UUS<br>A GAA AAG	20	UC{ UUS<br>A AGA AAG	5
UUS UUS<br>A AAG AAG	46	UUS CUS<br>A AAA GAG	0	UUS UUS<br>C AAA AAG	25	UGV UUS<br>A ACA AAG	22
UUQ UUS<br>A AAC AAG	5	UUS UUQ<br>A AAA AAC	9	UUS UUS<br>U AAA AAG	17	UA} UUS<br>A AUA AAG	29
UUQ UUS<br>A AAU AAG	25	UUS UAM<br>A AAA AUG	0	UUS UUQ<br>A AAA AAU	12	UUS UUS<br>G AAG AAG	0
UUS UUQ<br>A AAG AAU	3	UUS UUS<br>A AAG AAA	9	CC{ UUS<br>G GGA AAG	57	AA) UUS<br>U UUA AAG	39
UUS CC{<br>A AAG GGA	6						

Supplementary Table 4 Free-energy differences of base-pair changes. The mean μ and standard devitation σ for the free-energy difFSrences ΔG_{bp} that were obtained from the presented FS set (left table) and from the Tsuchihashi FS set (right table)[2].

Base-pair change(s) presented FS set	$\mu\,(\Delta G_{\mathrm{bp}})$ (kJ/mol)	$\sigma\,(\Delta G_{\mathrm{bp}})$ (kJ/mol)
P1 A·U→C·U	6.3	0.2
P1 A·U→U·U	7.5	0.2
P1 A·U→G·U	5.1	0.2
P1 U·A→A·A	2.3	0.1
P1 U·A→C·A	3.4	0.3
P1 U·A→G·A	2.7	0.3
A1 U·A→A·A	2.5	0.3
A1 A·U→U·U	6.8	0.2
A3 G·S→A·S	−2.9	0.1
A3 C·G→U·G	3.4	0.1
P3 G·S→A·S and A1 A·U→G·U	3.3	0.2
P3 G·S→A·S and A1 U·A→G·A	2.3	0.3
P3 C·G→U·G and A1 A·U→C·U	9.8	0.4
P3 C·G→U·G and A1 U·A→C·A	7.3	0.4

Base-pair change(s) Tsuchihashi FS set	$\mu\,(\Delta G_{\mathrm{bp}})$ (kJ/mol)	$\sigma\,(\Delta G_{\mathrm{bp}})$ (kJ/mol)
P1 A·U→C·U	7.6	1.2
P1 A·U→U·U	9.3	2.2
P1 A·U→G·U	8.6	1.4
P3 A·{→G·{	3.8	1.1
P3 A·)→U·)	5.7	1.1
A3 G·S→A·S	−4.6	0.9
P3 G·S→A·S and A1 A·U→G·U	5.2	0.9
P1 G·C→A·C and P2 A·U→G·U	8.5	1.4
P3 A·G→U·G and P2 U·A→A·A	7.0	1.2
P3 A·V→C·V and P2 C·G→A·G	8.1	1.3
P3 U·Q→A·Q and A1 A·U→U·U	7.5	1.2

Supplementary References

[1] Caliskan, N. *et al.* Conditional Switch between Frameshifting Regimes upon Translation of dnaX mRNA. *Molecular Cell* **66**, 558–567.e4 (2017).

[2] Tsuchihashi, Z. & Brown, P. O. Sequence requirements for efficient translational frameshifting in the Escherichia coli dnaX gene and the role of an unstable interaction between tRNALys and an AAG lysine codon. *Genes and Development* **6**, 511–519 (1992).

[3] Boccaletto, P. *et al.* MODOMICS: A database of RNA modification pathways. 2017 update. *Nucleic Acids Research* **46**, D303–D307 (2018).
