# Supplementary material for: Thermodynamic control of −1 programmed ribosomal frameshifting
Source: Nat Commun. 2019 Oct 10;10:4598. doi: 10.1038/s41467-019-12648-x (PMC6787027; doi:10.1038/s41467-019-12648-x)
Supplement: Supplementary file 2 — Peer Review [file 41467_2019_12648_MOESM2_ESM.pdf]

Reviewers' comments:

Reviewer #1 (Remarks to the Author):

In this manuscript, the authors ask whether thermodynamic considerations, alone, are sufficient to rationalize frameshifting efficiencies in the ribosome. To achieve this, the authors apply a Bayesian Inference approach to estimate the free energy of base pairing in the ribosome, using frameshifting efficiencies as a reference. Using the inferred free-energy differences, they then predict the frameshifting efficiencies obtained in a separate data set. Overall, this provides strong evidence that frameshifting is a thermodynamically controlled process, which is likely to be of significant interest and value to the ribosome community.

Suggested changes:

1) Using "FE" to describe frameshifting efficiency was a bit clumsy to read, since "free energy" is also a major term used in this manuscript. I had to keep reminding myself that FE was not referring to free energy. To avoid this issue with other readers, perhaps the notation E_fs (subscript fs) would be smoother.

2) page 1 "A steric hindrance downstream of the slippery site impedes". It would be helpful to clarify what region constitutes the steric hindrance.

3) Figure 2. It would be clearer if the caption title reads "Inferred mRNA-tRNA base-pair free-energy differences..." since one did not measure the differences directly.

4) Figure 6: It is confusing to have the labels "A site" and "P site" immediately above delta G_sol. Since the delta G _bp values correspond to energetics inferred on the ribosome, it would be more clear if the x axes were included the site labels.

5) Methods: Please provide complete details for how the MC search was performed. What was the effective potential and temperature for determining accepted moves? One could probably correctly guess, but explicitly stating these details will make it easier to reproduce.

Reviewer #2 (Remarks to the Author):

This manuscript is intended to demonstrate that the frequency of -1 programmed ribosomal frameshifting (-1 PRF) depends on the thermodynamic features of the base pairs formed during the process. The argument is largely theoretical and I am not able to affectively critique that work given my lack of background in the area. I can comment on the phenomenology of -1 PRF and on the accessibility of the theoretical argument to the largely non-expert audience of Nature Communications.

My major criticism of the manuscript is that it makes little effort to be understandable by these non-experts like myself. I do understand that the authors are expert in the field and I suspect, but cannot myself verify, that the arguments are sound. The manuscript, however, requires a rewrite that would make the argument more accessible. There are many places in the manuscript (arguably too frequent to cite) where the authors simply assert things that should be explained for these readers. The manuscript would be improved greatly by the authors making this attempt.

One part of the argument that seems to me to be circular is their description of their deriving the free energy of base pairs involved in various frameshift events then using those free energies to argue that the frameshifting can be explained by reference to the free energies of base pairing. It seems to me that if the free energies were derived by comparing the extent of frameshifting for the many

frameshifts tested then it would perhaps be inevitable that the free energies could then be used to justify the frameshift efficiencies. The authors do include a set of frameshifts that were not used to generate the free energy calculations but that test could only have been made if these second set of frameshifts involved base pairs that had not previously been tested. So, I would suggest that the authors need to be much more direct in explaining why this is not a critical flaw in their analysis. They may think that they have but in that case the attempt may have been to subtle for a non-expert to appreciate.

For free energy differences to explain frameshift behavior it would be necessary for the reaction to reach equilibrium. Efforts have been made by many researchers to determine the time scales of frameshift events but I have the impression that the elongation step at which frameshifting occurs cannot last more than seconds in vivo, certainly not approaching minutes. Would this be sufficient to achieve equilibrium? Perhaps the authors could address this more explicitly by referencing any data available on the kinetics of the reaction. This would make frameshifting different in kind from other translational events that kineticists like Dr. Rodnina have clearly shown to be kinetically and not thermodynamically regulated. The idea that the authors do not adequately make this argument comes from their statement on p. 4 (8 lines up) "assuming thermodynamic equilibration during the frameshifting"; surely this should not simply be assumed.

The idea that the stability or lack thereof of base pairs or mispairs formed during frameshifting help define the frameshift efficiency is not a new one. The value of this work seems to be that the authors provide a mathematical and theoretical model to explain that behavior. That is valuable on its face but I doubt it will change drastically our thinking about how these programmed errors occur.

There were issues with nomenclature of base pairing in several places. The nomenclature they proposes is in an X•Y base pair the X is a codon base and the Y anticodon. But they appear to violate that in several places. For example, in the last paragraph on p. 7 they suggest a change of base pairing during frameshifting from an A•U bp to a G•A or C•A bp, the latter which they describe as "purine•pyrimidine base pairs". These mistakes are probably typographical since the authors surely know the difference but it is disturbing to see these errors recur.

Finally, in describing base mispairs during frameshifting they refer to the work of the Marat Yusupov group (ref. 28) to support the idea that the ribosome tolerates wobble mispairing in the third codon position and that interactions in the first two positions "different" (top of p. 6) without discussing the A and P sites forcing U•G mismatches into a Watson-Crick conformation that is not allowed for other mismatches. Some consideration of how these new ideas about isostericity between canonical Watson-Crick pairs and some mispairs should be included in this work.

Thermodynamic Control of –1 Programmed Ribosomal Frameshifting
Reply to reviewer's comments

Reviewers' comments:

Reviewer #1 (Remarks to the Author):

In this manuscript, the authors ask whether thermodynamic considerations, alone, are sufficient to rationalize frameshifting efficiencies in the ribosome. To achieve this, the authors apply a Bayesian Inference approach to estimate the free energy of base pairing in the ribosome, using frameshifting efficiencies as a reference. Using the inferred free-energy differences, they then predict the frameshifting efficiencies obtained in a separate data set. Overall, this provides strong evidence that frameshifting is a thermodynamically controlled process, which is likely to be of significant interest and value to the ribosome community.

Suggested changes:

1) Using "FE" to describe frameshifting efficiency was a bit clumsy to read, since "free energy" is also a major term used in this manuscript. I had to keep reminding myself that FE was not referring to free energy. To avoid this issue with other readers, perhaps the notation E_fs (subscript fs) would be smoother.

We agree with the reviewer's comment that "FE" is easily confused with free energy. However, the E of E_fs would also suggest an energy. We therefore now define FS as frameshifting efficiency and changed it throughout the manuscript.

2) page 1 "A steric hindrance downstream of the slippery site impedes". It would be helpful to clarify what region constitutes the steric hindrance.

To clarify, we have changed the sentence to "The mRNA secondary structure element downstream of the slippery site impedes…"

3) Figure 2. It would be clearer if the caption title reads "Inferred mRNA-tRNA base-pair free-energy differences..." since one did not measure the differences directly.

We agree and changed the caption accordingly.

4) Figure 6: It is confusing to have the labels "A site" and "P site" immediately above delta G_sol. Since the delta G _bp values correspond to energetics inferred on the ribosome, it would be more clear if the x axes were included the site labels.

We agree and have moved the site label next to the G_bp label.

5) Methods: Please provide complete details for how the MC search was performed. What was the effective potential and temperature for determining accepted moves? One could probably correctly guess, but explicitly stating these details will make it easier to reproduce.

With equation 3 on page 14, we have obtained a function that is proportional to the probability distribution of P(Delta G_bp | FS_experiment) that we want to obtain. We used the Metropolis algorithm with this function to sample the unknown probability distribution. In this approach, neither a potential nor a temperature is required. We have now extended the description of the Metropolis sampling in the Methods text accordingly to clarify the procedure and to avoid misunderstandings.

The temperature *T* that is specified in the manuscript to calculate Boltzmann factors and frameshift probabilities is completely unrelated to the Metropolis sampling. We now have added the missing information that we used a temperature of T=310K.

Reviewer #2 (Remarks to the Author):

This manuscript is intended to demonstrate that the frequency of -1 programmed ribosomal frameshifting (-1 PRF) depends on the thermodynamic features of the base pairs formed during the process. The argument is largely theoretical and I am not able to affectively critique that work given my lack of background in the area. I can comment on the phenomenology of -1 PRF and on the accessibility of the theoretical argument to the largely non-expert audience of Nature Communications.

My major criticism of the manuscript is that it makes little effort to be understandable by these non-experts like myself. I do understand that the authors are expert in the field and I suspect, but cannot myself verify, that the arguments are sound. The manuscript, however, requires a rewrite that would make the argument more accessible. There are many places in the manuscript (arguably too frequent to cite) where the authors simply assert things that should be explained for these readers. The manuscript would be improved greatly by the authors making this attempt.

We thank the Referee for pointing this out. Motivated by the Referee's comment, we have revised the manuscript to make it better accessible to the broad audience of Nature Communications. We assume that the referee in particular refers to the (rather technical) description of the Bayes approach and the Monte Carlo Sampling. As the details of calculations are not essential for understanding the main idea, approach, or the results of the paper, we have now moved them to the Methods Section, which has improved the overall flow of the main text and – we hope – made the arguments more accessible. We also have added a few more general sentences to guide the reader through the main steps and conclusions.

One part of the argument that seems to me to be circular is their description of their deriving the free energy of base pairs involved in various frameshift events then using those free energies to argue that the frameshifting can be explained by reference to the free energies of base pairing. It seems to me that if the free energies were derived by comparing the extent of frameshifting for the many frameshifts tested then it would perhaps be inevitable that the free energies could then be used to justify the frameshift efficiencies. The authors do include a set of frameshifts that were not used to generate the free energy calculations but that test could only have been made if these second set of frameshifts involved base pairs that had not previously been tested. So, I would suggest that the authors need to be much

more direct in explaining why this is not a critical flaw in their analysis. They may think that they have but in that case the attempt may have been too subtle for a non-expert to appreciate.

The argument is in fact not circular and we have introduced changes that should clarify this issue. In short, our validation strategy is the following. We first use all efficiencies to calculate the base-pair free energies assuming the simple thermodynamic model and a limited number of independent parameters. The resulting values provide the best overall solution, but this does not automatically mean that each individual frameshifting value is faithfully reproduced. This is akin of analyzing residuals when fitting the data, which is a well-established method to validate the results of fitting.

Contrary to the expectation of the Referee, the good match in the experimental and fitted result is far from “inevitable”, because the number of measured frameshift efficiencies (64) is much larger that the number of independent parameters (14) in our free energy model. Furthermore, if the assumptions of our model were wrong, it would be highly unlikely that the few parameters can reproduce all 64 efficiencies. Using again the analogy to conventional fitting, if the model is incorrect, some points will be outside the fit. The result that the model is consistent with the data (Fig. 2d) suggests in particular that the underlying assumptions are also consistent with the measured frameshift efficiencies, namely (1) the assumption of equilibrium (see answer to the next point), and (2) that the base-pair free-energy differences are additive.

In the second step, as correctly pointed out by the Referee, we test whether or not the model has predictive power, i.e., if it is able to predict efficiencies that were not used to obtain the free-energy differences. For this purpose, we excluded one frameshifting value from the dataset, obtained the free-energy differences of our model from all other efficiencies and then tested how well these free-energy differences can predict the efficiency of the excluded mRNA variant. These steps were repeated for all mRNA variants. We obtained a good agreement with the measured efficiencies (Fig. 2e), underscoring that the model is indeed predictive. Note, however, that omitting all frameshift efficiencies involving a certain base pair, as suggested by the Referee, would not work: When we predict the efficiency of a certain mRNA variant from the base-pair free-energy differences, we need to obtain the free-energy differences involved in the frameshifting of this variant from the efficiencies of other variants that also involve this base-pair change. It is crucial to note that all the different variants lead to different combinations of base-pair changes, so there is no redundancy in the set of efficiencies which would preclude cross-validation.

We have now rephrased this part of the manuscript to make our approach and the motivation for the two steps more accessible.

For free energy differences to explain frameshift behavior it would be necessary for the reaction to reach equilibrium. Efforts have been made by many researchers to determine the time scales of frameshift events but I have the impression that the elongation step at which frameshifting occurs cannot last more than seconds in vivo, certainly not approaching minutes. Would this be sufficient to achieve equilibrium? Perhaps the authors could address this more explicitly by referencing any data available on the kinetics of the reaction. This would make frameshifting different in kind from other translational events that kineticists like

Dr. Rodnina have clearly shown to be kinetically and not thermodynamically regulated. The idea that the authors do not adequately make this argument comes from their statement on p. 4 (8 lines up) "assuming thermodynamic equilibration during the frameshifting"; surely this should not simply be assumed.

We thank the referee for bringing up this important point. In fact, translocation is usually a rapid process, which would preclude re-equilibration of tRNAs in a different reading frame while they translocate. However, the rate of translocation changes dramatically when the ribosome arrives at the slippery site followed by a downstream mRNA secondary structure element. The downstream mRNA structure slows down the completion of translocation, which may provide the time window for slippage. This is stated on pp. 1-2 and 6. Still, the question is whether the rate of frameshifting is sufficiently high compared to the completion of translocation. Because until recently there were no estimations of the intrinsic frameshifting rates, in this work we explicitly challenged the assumption that tRNA equilibrate during frameshifting (p. 6). The results of the calculation suggest that the contribution of the kinetic partitioning is negligible for the mRNA that has a dnaX mRNA secondary structure element. In the meantime, we were able to estimate the rates of frameshifting for the original dnaX slippery sequence and the A_4G mutant, 10 s^{-1} and 3 s^{-1} from 0- to –1-frame, respectively, compared to the rate of translocation of 0.1-0.5 s^{-1} in the presence of the hairpin. The manuscript in which the frameshifting rates are presented is submitted for publication; we attach the preprint for the Referee's peruse. This information is now included on p. 6, together with additional sentences that should clarify the kinetic argument. Further discussion on the limits for the thermodynamic model can be found in conclusions of this manuscript, p. 11, where we now also added a sentence for clarity.

The idea that the stability or lack thereof of base pairs or mispairs formed during frameshifting help define the frameshift efficiency is not a new one. The value of this work seems to be that the authors provide a mathematical and theoretical model to explain that behavior. That is valuable on its face but I doubt it will change drastically our thinking about how these programmed errors occur.

Indeed, the idea that the stability of base pairs influences frameshifting efficiencies is not new as such. In fact, this notion serves as an implicit guide to estimate whether an mRNA sequence is slippery or not. But exactly here is a problem exemplified by the high frameshifting efficiencies of A_1G and A_4G mutants (pp. 2 and 9), which at the first glance should not make frameshifting at all. Similarly, it is rather difficult to understand the effects of tRNA modifications without knowing the contributions of base pairing for modified tRNAs in the 0- and –1-frame. It is therefore essential to have a quantitative understanding of how the free-energy differences result in the efficiencies. This work provides not only a theoretical model, but also the first quantitative estimates for the interaction energies during frameshifting. This quantitative understanding makes it possible to predict the frameshifting efficiencies from the mRNA sequence and provides insights into the role of tRNA modifications. Thus, this work makes a crucial step from a qualitative (and disputable) notion to quantitative predictions that can be tested in further experiments.

There were issues with nomenclature of base pairing in several places. The nomenclature they proposes is in an X•Y base pair the X is a codon base and the Y anticodon. But they appear to violate that in several places. For example, in the last paragraph on p. 7 they

suggest a change of base pairing during frameshifting from an A•U bp to a G•A or C•A bp, the latter which they describe as "purine•pyrimidine base pairs". These mistakes are probably typographical since the authors surely know the difference but it is disturbing to see these errors recur.

There was indeed a typo in the referred sentence. In the bracket it should have said “(G•U or C•A)” instead of “(G•A or C•A)”. In describing these base pairs as purine•pyrimidine base pairs, we meant to say that this base pair consists of one purine and one pyrimidine without implying that the purine is the codon and the pyrimidine is the anticodon base. This description is in fact inconsistent with the nomenclature we used throughout the manuscript. We have changed the text to solve this problem (page 7, highlighted text) and thank the reviewer for noting the typo and the inconsistent nomenclature.

Finally, in describing base mispairs during frameshifting they refer to the work of the Marat Yusupov group (ref. 28) to support the idea that the ribosome tolerates wobble mispairing in the third codon position and that interactions in the first two positions "different" (top of p. 6) without discussing the A and P sites forcing U•G mismatches into a Watson-Crick conformation that is not allowed for other mismatches. Some consideration of how these new ideas about isostericity between canonical Watson-Crick pairs and some mispairs should be included in this work.

The reviewer refers to the sentence: “For all codons in the 0-frame, the 1st and 2nd positions of the codon-anticodon complex allow only Watson-Crick interactions, whereas in the 3rd position Watson-Crick and wobble base-pairs are tolerated[35,28].”
This sentence was meant to describe the codon-anticodon interactions in the 0-frame of the sequences used in this work, rather than to discuss how mismatches can be induced or tolerated by the ribosome. To avoid this misunderstanding, we have now changed the sentence and moved it to the place where we introduce the slippery sequences (page 4, highlighted).

Following the referee’s comment, we have now extended the discussion section by including the ideas about the isostericity of WC pairs and the U•G mismatches and the conclusions from a large set of x-ray structures of cognate and near-cognate tRNAs bound to 70S ribosomes (p. 11, highlighted).

REVIEWERS' COMMENTS:

Reviewer #1 (Remarks to the Author):

The authors have suitably address all questions.

As a note: In the original version, there was no confusion about which temperature was used for analysis. My comment regarding "effective temperature" was intended to ask which quantities were used to calculate the Metropolis acceptance criterion (e.g. delta_U -> change in scoring function/effective potential, kT -> 1)

Reviewer #2 (Remarks to the Author):

I believe that the manuscript is much improved by the revisions and now have no criticisms to offer.

I thank the authors for taking my possibly too harsh comments with good grace. I do believe, however, that the result of their revision is a manuscript that will be much more accessible to a general audience, which could have the beneficial effect of more broadly disseminating their results.
